# Black Sea hydrate production value and options for clean energy production

**DOI:** 10.1039/d3ra03774f

**Published:** 2023-07-11

**Authors:** Bjørn Kvamme, Atanas Vasilev

**Affiliations:** a State Key Laboratory of Oil and Gas Reservoir Geology and Exploitation, Southwest Petroleum University Xindu Road No. 8 Chengdu 610500 China bkvamme@strategic-carbonllc.com +86 47 9345 1956; b State Key Laboratory of Natural Gas Hydrate Sun Palace South Street No. 6 Beijing 10027 China; c Strategic Carbon LLC 7625 Rancho Vista BLVD W Corpus Christi 78414 TX USA; d Institute of Oceanology – Bulgarian Academy of Sciences First May str. 40, PO Box 152 Varna 9000 Bulgaria

## Abstract

Natural gas hydrates of Bulgaria and Romania in the Black Sea have been subject to studies by several European research projects. The current understanding of the hydrate distribution, and the total amounts of hydrate in the region, makes it interesting to evaluate in terms of commercial potential. In this study, we have evaluated some well-known hydrate production methods. Thermal stimulation and adding chemicals are considered as not economically feasible. Pressure reduction may not be efficient due to the endothermic dissociation of hydrates and long-term cooling of the sediments. Chemical work due to pressure reduction is an additional mechanism but is too slow to be commercially feasible. Adding CO_2_/N_2_, however, has a dual value. In the future, CO_2_ can be stored at a price proportional to a CO_2_ tax. This is deducted from the value of the released natural gas. The maximum addition of N_2_ is around 30 mol% of the CO_2_/N_2_ mixture. A minor addition (in the order of 1 mol%) of CH_4_ increases the stability of the hydrate created from the injection gas. The maximum N_2_ amount is dictated by the demand for the creation of a new hydrate from injection gas but also the need for sufficient heat release from this hydrate formation to dissociate the *in situ* CH_4_ hydrates. An additional additive is needed to accelerate the formation of hydrate from injection gas while at the same time reducing the creation of blocking hydrate films. Based on reasonable assumptions and approximations as used in a verified kinetic model it is found that CH_4_/CO_2_ swapping is a feasible method for Black Sea hydrates. It is also argued that the technology is essentially conventional petroleum technology combined with learning from projects on aquifer storage of CO_2_, and a thermodynamic approach for design of appropriate injection gas. It is also argued that the CH_4_/CO_2_ swap can be combined with well-known technology for steam cracking of produced hydrocarbons to H_2_ and CO_2_ (for re-injection).

## Abstract 2

Natural gas hydrates of Bulgaria and Romania in the Black Sea have been subject to studies by several European research projects.

## Graphical abstract

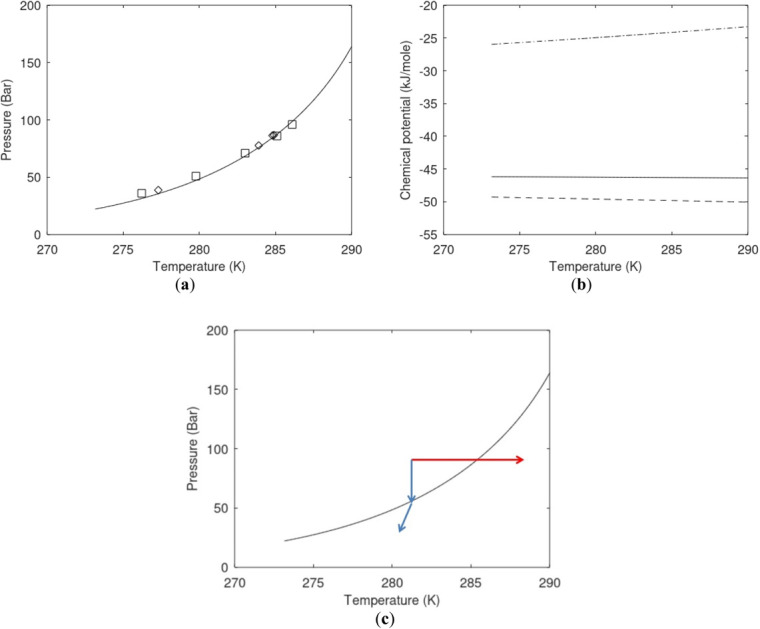

## 1. Introduction

Even more than 20 years ago it was estimated^1^ that the energy in the form of hydrate is more than twice the known amounts of all known sources of conventional fossil fuels, including coal. Hydrates are widely spread globally and if natural gas in them replaces some coal that would be a first environmental step. The potential of a gas hydrate production cycle with H_2_ as the only net energy export product^2^ is even more attractive and a big step forward. Technology solutions for the commercial use of H_2_ in the private energy sector, as well as for use in the public transport sector, have been available for many years already.

Comparing worldwide hydrates to shale gas raises some perspectives, in addition to the abundance of natural gas hydrates. Fracking shale gas deposits are costly and involve various kinds of risks. Risk of geo-mechanical instabilities, undesired gas leakage pathways, and the use of chemicals including toxic are just three examples. The paradox is that the production of natural gas from hydrates may be simpler than shale gas production, and involve less risk. Some disagree with this statement and it is timely to have an open debate on this.

**Fig. 1:**
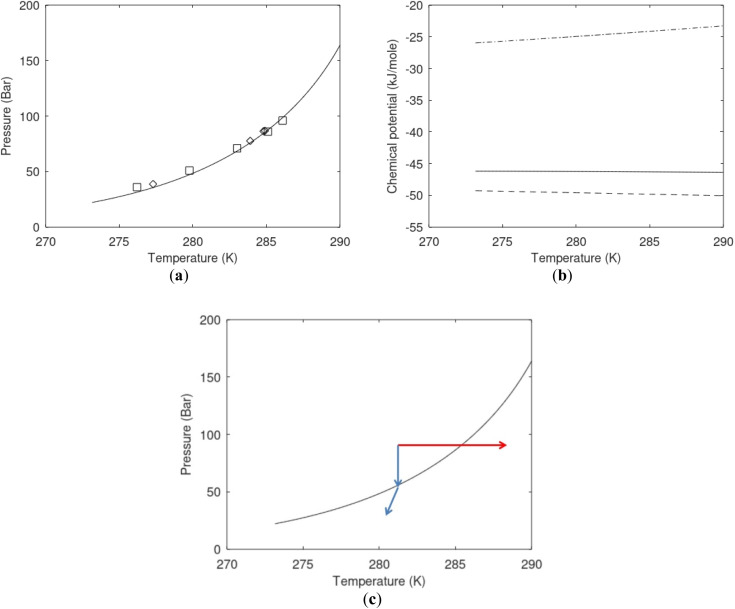
(a) Pressure temperature hydrate stability limits for CH_4_ hydrate. Solid is calculated (equations in Section 3), squares are experimental values from Sabil *et al.*^13^ and diamonds are experimental data from Tumba *et al.*^14^ (b) Hydrate Gibbs free energy (solid), liquid water chemical potential (dashed), and CH_4_ gas chemical potential (dash-dot). (c) Qualitative illustration of thermal stimulation for hydrate dissociation (red) and qualitative illustration of pressure reduction for hydrate dissociation (blue). The Joule–Thomson cooling due to gas expansion from the hydrate stability curve is of course not a straight line so just qualitative illustration.

One of the limitations of the discussion of different methods for dissociating hydrates and releasing gas today is the thermodynamic analysis. A rigorous analysis based on the fundamental laws of thermodynamics is mostly absent. Instead, various production methods are evaluated based on simplified projections of hydrate stability like the one in Fig. 1c. It is not random that these curves are named stability limits rather than equilibrium curves. It is well known to most hydrate researchers that two components (one guest and water) distributed over three phases can establish thermodynamic equilibrium if one, and only one, independent thermodynamic variable is defined.^1,3^ This can easily be seen from Gibbs phase rule, which ends up with one degree of freedom for 2 components distributed over 3 phases (gas, liquid water, hydrate). Systems containing water in porous media are fairly complex. One reason is the strong interactions between water and mineral surfaces. Another reason is the interfaces between hydrates and liquid water. Yet a third reason is the selective adsorption of guest molecules (hydrocarbons, CO_2_, H_2_S, N_2_*etc*) on liquid water surface. These phases are not massive in terms of mole numbers but still thermodynamically important. Many phases that will normally not be considered in analysis of conventional hydrocarbon systems play important roles. Instead of the compact Gibbs phase rule it might be better to use the rigorous counting behind the simplified rule. Degrees of freedom are number of independent thermodynamic variables minus constraints minus constraints on these variables. Constraints are conservation laws and thermodynamic equilibrium equations. Selective adsorption on the liquid water interface gives rice many different hydrates from mixtures. Kvamme^4^ illustrated this using a simple 2D adsorption theory to calculate adsorbed mole fractions of CO_2_ and N_2_ on liquid water surface *versus* composition in gas. Hydrate nucleation is thermodynamically and kinetically favoured in the liquid water side of the interface.^5–7^ See also all the papers included in several PhD theses on mesoscale modelling of hydrate phase transitions.^8–11^ There is nothing new about the one degree of freedom for three phases (liquid water, gas, hydrate) and two components (liquid water and CH_4_ for example). Experimentalists of hydrate equilibrium back in the 1940s used various methods to control pressure (*P*) or temperature (*T*) and measure the other one. One way was to fix pressure and form the hydrate at some temperature. Then, still, at the same pressure, they slowly increased temperature until the hydrate started to dissociate. The use of stirring at different levels made the formed hydrate more or less homogeneous, on a macroscopic level at least, so the range of hydrates we might expect from a mixture in a system without imposed hydrodynamics is not visible. Experimental data for hydrate equilibrium back to the early experiment has been compiled and reported by Sloan and Koh.^12^

When two independent variables are defined by nature, like in natural sediment containing hydrates, then equilibrium equations are no longer valid. It is a mathematical consequence that the number of constraints (conservation laws and equilibrium equations) and fixed thermodynamic variables (*T* and *P*) exceeds the number of independent variables. Then there is no unique mathematical solution. One consequence of this is that there will be competing phase transitions that can result in hydrate formation and hydrate dissociation. Frequently thermal and mechanical equilibrium can be reached but then the chemical potentials of each component cannot be the same across phase boundaries.^3^ A degree of freedom of 1, which is the number of independent variables that must be defined for equilibrium, is even high for hydrates in sediments. Minerals surfaces are active as both inhibitors and promoters.^4^ Mixtures of guest molecules make the non-equilibrium situation more complex due to selective adsorption on liquid water and mineral surfaces (polar guests) and the creation of several hydrate phases. Throughout this paper, we will therefore use the term stability limits in different projections. Temperature and pressure are two variables defining one 2D projection. Hydrate stability limit as function of guest molecule concentration in surrounding water and temperature and pressure is another 3D projection (see Fig. 4 in Section 3). A third one is the hydrate stability limit in a concentration of water in the guest phase. The list is long. There is nothing like quasi-equilibrium, locally in the pores there are, however, some phase transitions that may be more dominating than others for specific times. And it is all kinetics in a thermodynamically non-equilibrium system.

The problem with the evaluation of the hydrate production strategy based on Fig. 1c is that at least two important aspects are missing. Gibbs free energy change for hydrate formation, and hydrate dissociation, is zero for every point along the solid curve. Since our model is based on chemical potentials for all components the comparison with experimental data is model verification for the chemical potentials of water and guest. Chemical potentials are plotted in Fig. 1b together with Gibbs free energy for the hydrate. As long as thermal stimulation (red, Fig. 1c), or pressure reduction (blue), brings the system out of the solid hydrate stability limit curve the dissociated products (liquid water and CH_4_ gas) are more stable (lower Gibbs free energy) than the same amounts of the components collected into solid hydrate. As such the combined first and second laws of thermodynamics are satisfied. What is missing is whether the first law can be satisfied or not. Can the dissociation enthalpy be supplied and how? For thermal stimulation, the dissociation enthalpy is supplied directly through the heat in the form of steam, hot water, or some other thermal source. And finally what about the second law of thermodynamics? Dissociation of hydrate requires an entropy increase corresponding to breaking a solid hydrate structure over to structures of higher entropy, liquid water, and gas respectively. Is the level of temperature high enough to accomplish these entropy changes?

The discussion above illustrates part of the objectives of this work. From the open literature, there have been substantial challenges with the pressure reduction method. The first hydrate production test offshore Japan was planned for two weeks and was shut down after 6 days. Preliminary results were presented in a workshop in Kyoto in 2014 (Nanotechnology & Nano-geoscience in Oil and Gas Industry, Society of Petroleum Engineers, March 4–7, 2014, Kyoto, Japan) but results were fairly non-conclusive although sand and tendencies of freezing down were mentioned as some of the problems. The qualitative nature of the presentation is one of the reasons that the presentation is not listed among the references. Presentation was given by an assistant professor from Kyoto University. Sand production is highlighted as the major problem in a written report.^15^ The second pilot offshore Japan was planned for a test production period of 6 months.^16^ After 24 days of oscillating production rates, and indications of partial freezing down, the production stopped.^17^ In particular one of the powerpoint-slides discussed by Dr Tenma^17^ illustrated very clearly the periodic tendencies of freezing down in terms of very sharp reduction in released gas rates. Other problems were also discussed but it was still concluded that the test ended due to problems of freezing down. Heat transport through water phases is very fast compared to diffusional transport of molecules. Even though heat transport through hydrate is significantly slower than transport through liquid water heat transport through hydrate is still a fast molecular transport mechanism. Transport of solid particles (sand and hydrate), on the other hand, is on a hydrodynamic level and far slower. The very sharp, and also smooth, reduction in gas release in the power-point slide shown to the audience^17^ by Dr Tenma would be more in accordance with a freezing down scenario. Hydrodynamic controlled blocking caused by sand accumulations would be expected to show more irregular drops in gas release since it is unlikely that sand blocking occur simultaneously and rapidly over substantial regions of the gas release front. The discussion above does not exclude combinations of the two effects although Dr Tenma's explanation of freezing down as the mechanism that finally stopped the flow seems reasonable in view of his graph of the pressure *versus* time-line.

The main objective of this work is to illustrate how the laws of thermodynamics can analyse the feasibility of various production methods and minimize their values.

**Fig. 2:**
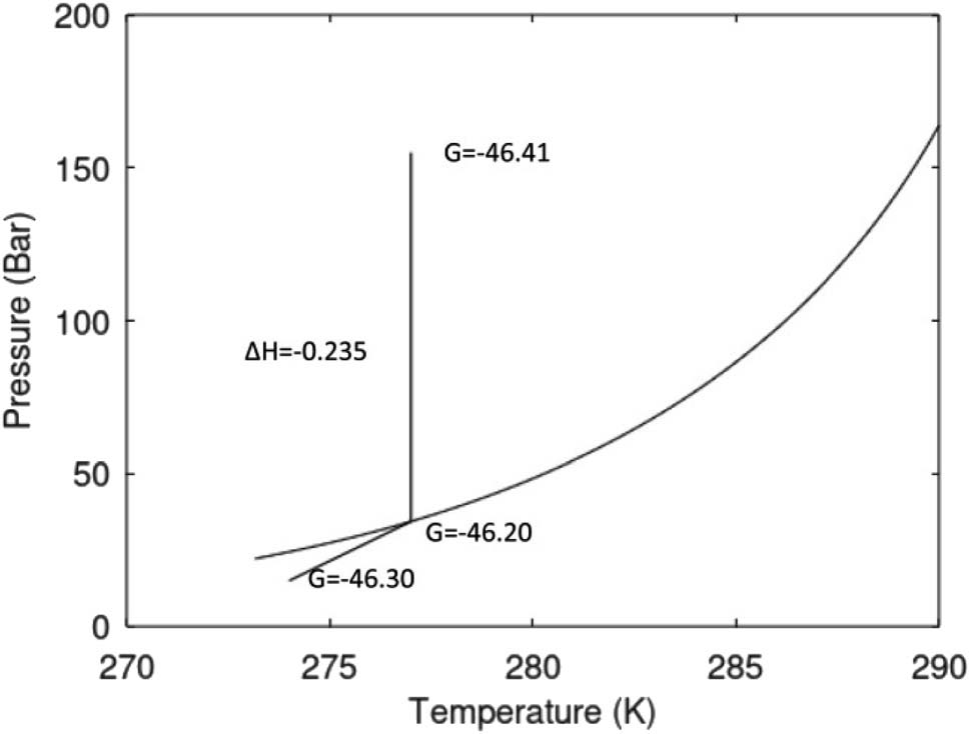
Pressure temperature hydrate stability limit curve as in Fig. 1a along with a first effect of pressure reduction to hydrate dissociation point at *P* = 34.43 bar for 277 K. Initial condition is 277 K and 155 bar. All Gibbs free energies (G) are in kJ mol^−1^ hydrate. For the final point Gibbs free energy is for the same amounts of water and gas that was in the hydrate at the dissociation point, but now in the form of liquid water and gas. The enthalpy change (Δ*H*) for the first pressure reduction is in kJ mol^−1^ hydrate for comparison with G in terms of units. The largest change in enthalpy is the dissociation enthalpy and dissociation condition of 277 K and 34.43 bar which is −7.57 kJ mol^−1^ hydrate (−56.24 kJ mol^−1^ CH_4_). The released CH_4_ gas from the initial state down to dissociation is 601 moles CH_4_ m^−3^ hydrate due to the rearrangement of the hydrate towards a lower filling fraction at the dissociation point.

A secondary objective is to illustrate the benefits of a uniform reference state for all phases. Ideal gas as reference state for all components in all phases ensures comparable Gibbs free energies for all possible phases in terms of stability. Derivation of the enthalpy model from the Gibbs free energy model using fundamental thermodynamic relationships ensures consistent entropy calculations for use in second law analysis. A simultaneous transition from fugacity description of guest molecules over to chemical potentials opens up a more general formalism that can be used in the analysis of hydrate formation and dissociation along a variety of pathways which also includes solid surfaces. A more specific example of the qualitative changes in Fig. 1c is plotted in Fig. 2. Initial CH_4_ hydrate at 277 K and 155 bar is exposed to a pressure reduction down to 15 bar. The final temperature in the figure is a function of the Joule–Thomson. A straight line down to 274 K is random for illustration only. The residual thermodynamic equations have been derived and presented in a substantial number of papers and we will mostly refer to these for detailed derivations and complete sets of equations.

The third objective is to utilize the thermodynamic analysis on a specific example from the Black Sea as part of a hydrate energy value analysis.

The fourth objective is to illustrate how the production of natural gas from hydrate can be turned into a concept that implicitly involves safe long terms storage of CO_2_ as solid hydrate, the fifth and final objective is to illustrate that the commercial production of natural gas from hydrate using CO_2_/N_2_ mixtures with extra additives is possible and well-known technology can be utilized. There are remaining research challenges toward higher production efficiency but this does not affect the main technology components of the production.

Finally there is also a fifth objective which is not devoted much attention in the paper simply because it is an optional add-on to the CH_4_/CO_2_ swap method. This add-on is based on well-established technology which was first developed in Norway in 1913. CH_4_ can be cracked by steam to H_2_ and CO_2_. Ideally one mole of produced CH_4_ from hydrate can be cracked to 2 moles H_2_ and 1 mole CO_2_. The CO_2_ cane be re-injected into the natural gas hydrate so that the only net delivery is H_2_. Some limited reflections on that can be found in Section 7.4.

This is not a review paper on methods to release natural gas from hydrates. It is mainly a thermodynamic paper intended to illustrate a methodology for the analysis of methods to release natural gas from hydrates. For this reason, there are no references to specific methods for adding heat in thermal stimulation or references to the experiments on pressure reduction. The aim is to propose sets of thermodynamic calculations before designing experiments, or pilots, for testing a specific hydrate production method. Systematic thermodynamic analysis, in which all the thermodynamic laws have specific implications for the feasibility of a specific production method, is the goal. This type of analysis can assist in the design of experiments and can be used to select the most appropriate hydrate production method for a given reservoir containing *in situ* natural gas hydrate. The novelty of this work is that this type of systematic thermodynamic analysis has never been published by any other research groups. Given the costs of pilot experiments and cost of large scale experiments the potential economic savings in avoiding tests that have little or no potential of succeeding is substantial. One example is already given by Kvamme^4^ in a more limited analysis of the Ignik Sikumi pilot on CO_2_ injection. The thermodynamic scheme for feasibility analysis utilized here is, however, more extensive and complete than the analysis of Kvamme.^4^

One challenge related to the objectives above is that there is only one available concept that is able to calculate all the properties needed in a consistent way. Residual thermodynamics for all phases is a necessity for comparing thermodynamic stability of co-existing phases. Enthalpy calculations that are consistent with Gibbs free energy calculations are necessary to ensure realistic calculations of entropy changes for hydrate phase transitions. In a simple analogy to dissociation of ice we simply need to ensure that the final entropy resembles the structure of liquid water. There is not even a simple way to utilize Clausius–Clapeyron equation through derivation from Gibbs–Duhem because of the complex relationships between chemical potentials of the different components in hydrate, and in the co-existing fluid mixtures. We have not found any appropriate derivation in open literature, even for binary hydrates. And the definition of fugacity for hydrate formed from a mixture is totally empirical and thermodynamically inconsistent. Fugacity is defined on a component basis. Derivation based on the fundamental relationship between fugacity and chemical potential results in fairly complex hydrate “fugacity”. This has been demonstrated by Kvamme in some earlier publications. The fact that there are many self-references is simply that we are the only one that use residual thermodynamics for all phases, and all thermodynamic properties needed. There are no other available consistent thermodynamic concepts to compare to. To our knowledge all other thermodynamic codes, academic and industrial, are based on methods from before 1970. In these approaches the chemical potential difference between pure liquid water and empty hydrate is empirically fitted. In order to correct for temperature and pressure changes of this chemical potential difference then also involves empirical fittings of similar enthalpy differences, specific heat capacity differences and molar volume differences.

There are 24 citations (of 95 references) with Kvamme as first author, and some additional references from collaborators and previous students.

## 2. Thermodynamic laws and impact on hydrate production schemes

The first law of thermodynamics for open systems in Legendre transformed version, with *P* as independent thermodynamic variable instead of volume, can be written as follows for *p* co-existing phases:1
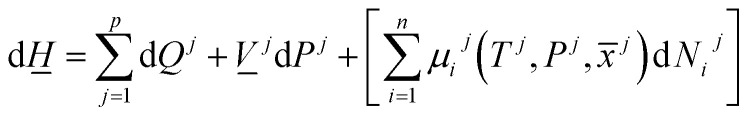
*H* is enthalpy and line below denotes extensive enthalpy in Joules. *Q*^*j*^ is added heat to phase *j* from external sources and other phases. μ^*j*^_*i*_(*T*_*j*_,*P*_*j*_,*x̄*_*j*_) is the chemical potential for component I in phase *j*. As an example, consider p equal to 3 with *j* = gas, liquid water or hydrate. Minerals are considered as external and initially adsorbed phases on minerals are skipped. For heterogeneous hydrate formation on gas/liquid water interface there is one temperature acting from gas side, and potentially there is a different temperature acting from liquid water side. Heat transport is very fast through water phases, as compared to heat transport through non-polar gas (or liquid), even though conductivity through hydrate is lower than heat conductivity through liquid water.2
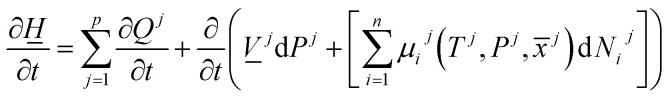


The heat transport related to hydrates in sediments under dynamic situations is mainly due to molecular transport in the form of heat conduction, and also by heat convection. Complex and detailed formulations are not needed for the purpose of this work. The following expressions for heat conduction in direction *x*, based on volumetric description in *x*, *y* and *z* directions, can be expressed as:3
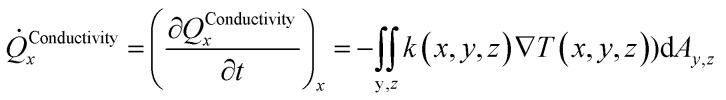
*k* is the heat conductivity. Inside the “bulk” of a phase *j* it is uniform in all three directions and more complex across interfaces between different phases. The units of *k* is (J m^−2^ K^−1^ s^−1^). Interface between hydrate and liquid water is a roughly 1.2 nm thick region^10,11,18,19^ in which water is gradually more structured from liquid water side towards the hydrate side. Density of water goes down and then also the heat conductivity is reduced from liquid water to hydrate. Another example is interface between liquid water and gas which can be very complex in other ways. Capillary waves and other interfacial phenomena make an equation like (3) over-simplified. For our purpose, however, the details are not important. The connections to the thermodynamic laws, and relationships between level of temperature and thermodynamic functions are the primary focus here. ∇*T* are the three-dimensional gradients in temperature. d*A*_*y*,*z*_ is the differential of the surface perpendicular to direction *x* (*y* and *z* are orthonormal to *x*). It is not ideal to use *x* for geometric direction, and for mole-fraction in thermodynamic equations. Heat conduction is by definition never truly zero since temperatures vary proportional to dynamic variations in molecular momentums. On a macroscopic scale, however, there is no average heat conduction in a multiphase system of uniform temperatures.

The heat convection can be expressed as:

4 *Q̇*^Convection^_*x*_ = *h*(*x*,*y*,*z*)*A*_*y*,*z*_(*T*_*x*,0_ − *T*_*x*_)

*h* is the heat transfer coefficient in units (J m^−2^ K^−1^ s^−1^). Subscript _*x*,0_ in eqn (4) denote a temperature at a reference point like for instance a solid surface or an interface between two phases. Subscript *x* denote temperature in a certain position in *x*-direction from the reference position. Convection is macroscopically defined and by definition 0 inside the bulk of a uniform phase.

The role of the first laws of thermodynamics is in the energy balances, whether that is in the form of mathematical models inside a reservoir simulator, or as part of a thermodynamic analysis as in this work.

The second law of thermodynamics for the same system can be written as:5
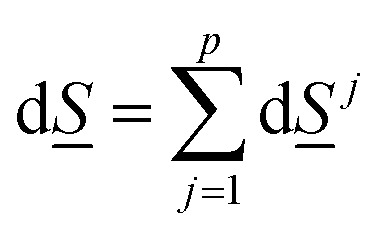


The entropy development for every phase inside the sum of eqn (5) is on the form of:6
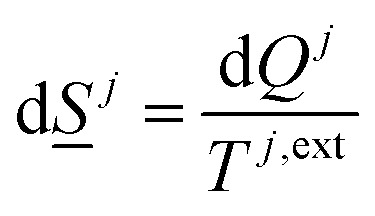


The superscript *j*,ext in eqn (6) involves temperatures from external sources but also from other phases with different temperatures. The second part of the second law is that heat cannot be transported directly from a lower temperature to a higher temperature.

On a simple first order analysis, on the same level as eqn (2), we can then write:7
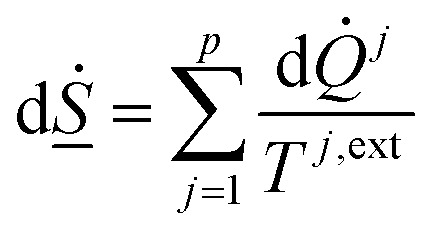


A direct role of the second law is the control over level of temperature resulting from various stimulations that can be applied with the intention to dissociate hydrate. The actual temperature that faces the hydrate, and then also the interface between hydrate and liquid water, has to be sufficiently high to break the hydrogen bonds. Entropy development during these changes should result in structure of liquid water and structure of gas from guest molecules after hydrate dissociation. Thermodynamically Helmholtz free energy is directly linked to the structure through the canonical ensemble, which again links to Gibbs free energy by a trivial Legendre transform. Enthalpy is fundamentally linked to Gibbs free energy (eqn (16) in Section 3). Definition of Gibbs free energy as function of enthalpy and entropy is the reason why the models for Gibbs free energy and enthalpy need to be consistent and based on the same model, as a minimum requirements for prediction of realistic structural changes, and entropy changes, during phase transitions.

**Fig. 3:**
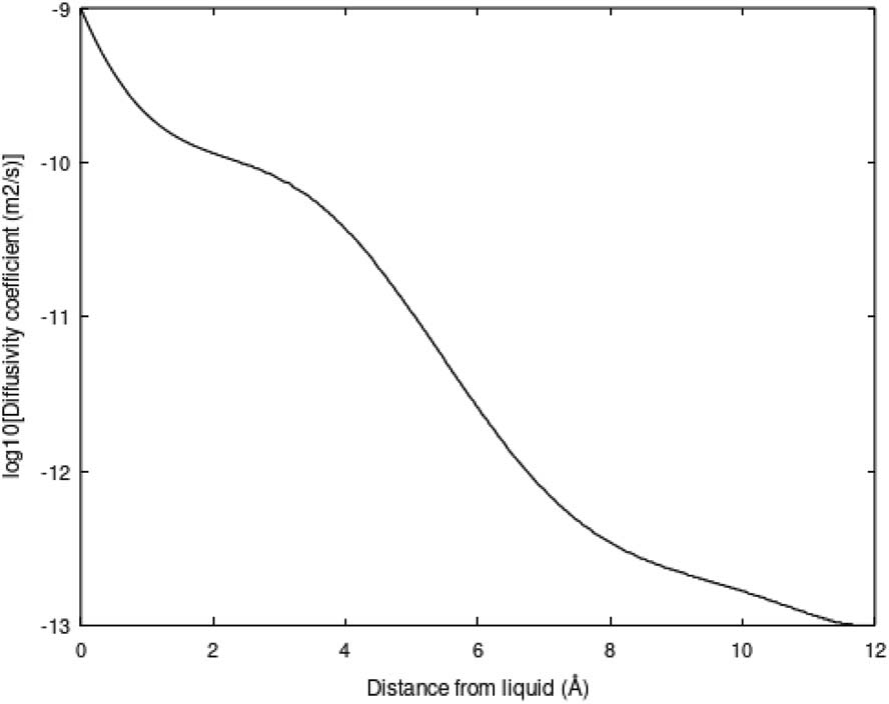
Diffusivity coefficients of water in m^2^ s^−1^ as plotted logarithmic scale as function of distance from liquid side of interface in Ångstrøms. Diffusivity coefficient on liquid water interface is 0.99 × 10^−9^ m^2^ s^−1^ and diffusivity coefficient towards hydrate side is 1.01 × 10^−13^ m^2^ s^−1^.

Molecular diffusion changes dramatically during hydrate dissociation. The partial molar entropy for water changes from a low entropy in hydrate to a significantly higher value in liquid water. The entropy partial molar entropy change for the guest molecules, on the hand, depends on the dissociation route. If hydrate dissociates over to gas the partial molar entropy change can be large, in contrast to dissociation of hydrate towards liquid water. In the latter case the guest is then initially transferred to liquid water solution. Frequently the average structure of guest molecules in liquid water are similar to structure in hydrate, like for instance CH_4_ in small cavity transferred to liquid water solution. Hydrate stability limits towards liquid water is illustrated in figure in Section 3. Fig. 3 below is a diffusivity coefficient profile based on the simulation methods due to Kvamme & Tanaka.^20^ The uncertainty in diffusivity coefficient is large and the profile might very well be considered as semi-empirical. The interface thickness is, however, derived from other types of molecular dynamics simulations.^10,18,19^ The diffusivity profile appears very reasonable relative to the structural development of water across the interface (1.2 nm). See also Kvamme *et al.*^21^ for prediction of hydrate film thickness as function of time as compared to experimental data for CH_4_ hydrate and CO_2_ hydrate, in which this type of interface diffusivity coefficient profile was utilized.

The diffusivity coefficient on the hydrate surface is expected to be substantially higher than water diffusivities inside “bulk hydrate”. Estimates of “bulk hydrate” water diffusivity coefficients may be in the order of 10^−15^ m^2^ s^−1^.^22–24^ Estimates and even methods for calculations of “bulk hydrate” diffusivity are, however, very uncertain.

This interface is a physical necessity for co-existence between hydrate and liquid water and stems from the fact that partial charge distribution from hydrate surface waters is fairly fixed geometrically and does not match partial charges in average dynamic structures of liquid water. The interface reproduces itself continuously on a molecular scale in time and space. Practically this is a kinetic bottleneck in hydrate dissociation kinetics. Any efficient hydrate production scheme need to deliver temperatures high enough to break the hydrogen bonds in the hydrate/water interface, as well as in “bulk hydrate”. This is a substantial challenge for the pressure reduction method. The impact of the hydrogen bond structure on hydrate phase transition kinetics is more complex than just the slow mass transport across the interface diffusivity profiles like the one in Fig. 3. Concentration profiles of the guest molecules across the interface are fairly steep from concentrations close to solubility on the liquid water side to high concentration on the hydrate side of the interface. See for instance Kvamme *et al.*^21^ for more details.

The combination of first and second laws of thermodynamics expressed in the same independent variables (temperatures, pressures and mole-fractions) as the first law, and expressed in terms of Gibbs free energy is:8
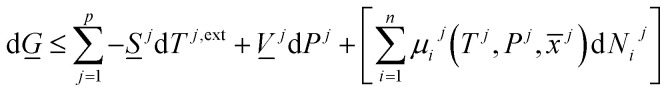
in which the line below *G* denote extensive property (unit Joule), *j* is a phase index and *N* is the number of moles in each phase. Total number of co-existing phases is *p*. Line under *S* and *V* denotes extensive properties with SI units J K^−1^ and m^3^ respectively. Superscript *j*,ext means the temperature acting on phase *j* from surroundings.

There are two primary roles of the combined law for the systems in this work. For systems that can reach thermodynamic equilibrium then eqn (8) can be used to derive equilibrium conditions. That part of it can, however, also be directly and easier derived from eqn (1) when Legendre transformed back to internal energy formulation rather than enthalpy formulations. The second and most important role of the combined law in this work is thermodynamic directions for phase separations and creation of new phases. In first order dynamic form the result is:9
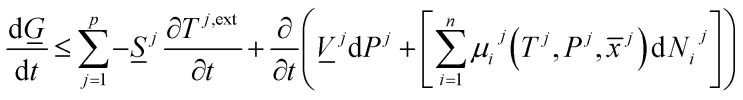


## 3. Residual thermodynamics

**Fig. 4:**
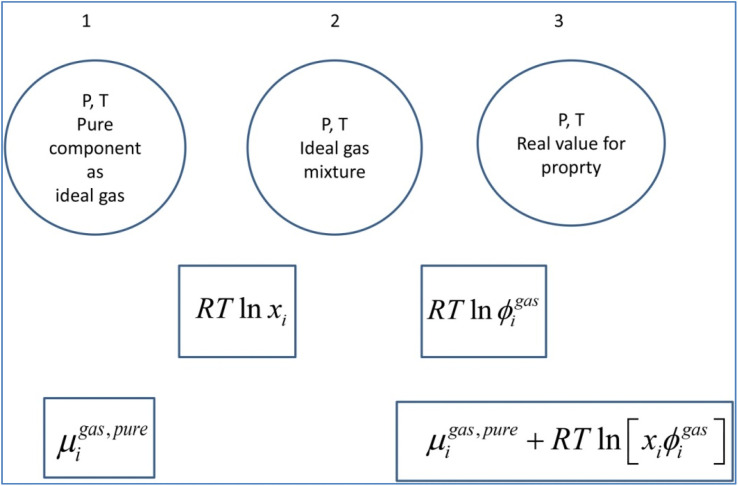
Residual thermodynamic scheme for guest molecule chemical potential in fluid phase. Chemical potential for pure component as ideal gas is trivially available from the canonical partition function for the given molecule type as function of molecular mass (translational momentums) and moments of inertia for molecules with rotational degrees of freedom. All molecules in this study are rigid molecules based on successful models applied in several Molecular Dynamics (MD) studies. Methane is approximated as a simple sphere. Moments of inertia for CO_2_ and H_2_O (TIP4P^31^) are available from the authors. Ideal mixing from 1 to 2 comes from the entropy effect of dilution when pure components mix at constant *P*. The fugacity coefficient goes to unity when pressure goes to zero. For other conditions it can trivially be derived from Helmholtz free energy for any equation of state, we use Soave–Redlich–Kwong (SRK) equation of state.^32^ Analytical expression for the fugacity coefficient from SRK is available from several chemical engineering handbooks. Pure water chemical potential as ice were sampled from MD studies, in which the ideal gas chemical potentials are sampled from the momentum space and the residual contribution (corresponding to the step from 2 to 3) are sampled from configurational space.^20^ Liquid water chemical potentials are derived used experimental data for dissociation of ice and liquid water heat capacities.^20^ For water containing salts or other dissolved components then a symmetric excess scheme applies. In this case pure also three steps apply. Reference state similar to 1 in this figure is now pure liquid water. From 1 to 2 an ideal mixing term is added similar to the residual scheme. The result at 2 is ideal mixture for water and then the correction from 2 to 3 is accomplished by replacing the fugacity coefficient with an activity coefficient that accounts for dissolved molecules (and ions) effects on water chemical potential. Symmetric activity coefficient goes to unity when ole-fraction water goes to unity. A similar three stage approach also applies to guest molecules dissolved in water. Due to it’s quadrupole moment CO_2_ is more soluble in water than CH_4_. Solubility of these components in water is still low and infinite dilution of these components in water is a proper reference state. Reference stage is 1 in this figure will now the infinite dilution chemical potential for the actual component in water, which is available from MD simulations for model molecules but can also be derived from experimental data. The same ideal mixture term from 1 to 2 apply. And then there is an asymmetric excess activity coefficient that replaces the fugacity coefficient in the step from 2 to 3. Asymmetric activity coefficient approaches unity when mole-fraction of the dissolved guest in water is zero.

A general residual thermodynamic scheme is illustrated in Fig. 4.

The detailed equations for different components in the different phases are available from several references. Ref. 1, 25–30 are some examples.

Chemical potential for water in hydrate is given by:^10^10
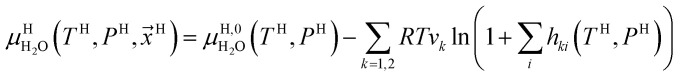


*μ*_H_2__^H,0^_O_(*T*^H^,*P*^H^) is chemical potential for water in empty hydrate from Kvamme and Tanaka^19^ and corrected by a simple Poynting correction from 1 bar to actual pressure of the hydrate *P*^H^. *v*_*k*_is the number of cavities of type *k* per water molecule. In structure I hydrates, which we use in this work as examples, there are 6 large cavities and 2 small cavities in a cubic water structure of 46 water molecules. *v*_*k*_is 3/23 for *k* = large and 1/23 for *k* = small. The canonical partition function for molecule *i* in cavity type *k* can be written as:

11 *h*_*ki*_ = e^*β*[*μ*_*ki*_−Δ*g*_*ki*_]^

*μ*_*ki*_is chemical potential for molecule type *i* in cavity type *k*. Δ*g*_*ki*_ is the free energy of inclusion of the guest molecule *i* in cavity type *k* as sampled from MD. Functions for free energy of inclusions as function of temperature are available elsewhere.^1,20,21,25–30^

Assuming that small and large cavities are at equilibrium:

12 *μ*_large_*i*__ = *μ*_small_*i*__

Eqn (10) is derived from a semi grand canonical ensemble in which number of water is fixed and number of guest molecules corresponds to an open ensemble.

Fig. 1a is constructed based on equal chemical potentials for liquid water and hydrate water as in an equilibrium system but now denoted as stability limits. Mechanical and thermal equilibrium implies that phase indexes can be omitted on these and we end up with:13
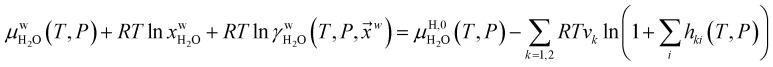


**Fig. 5:**
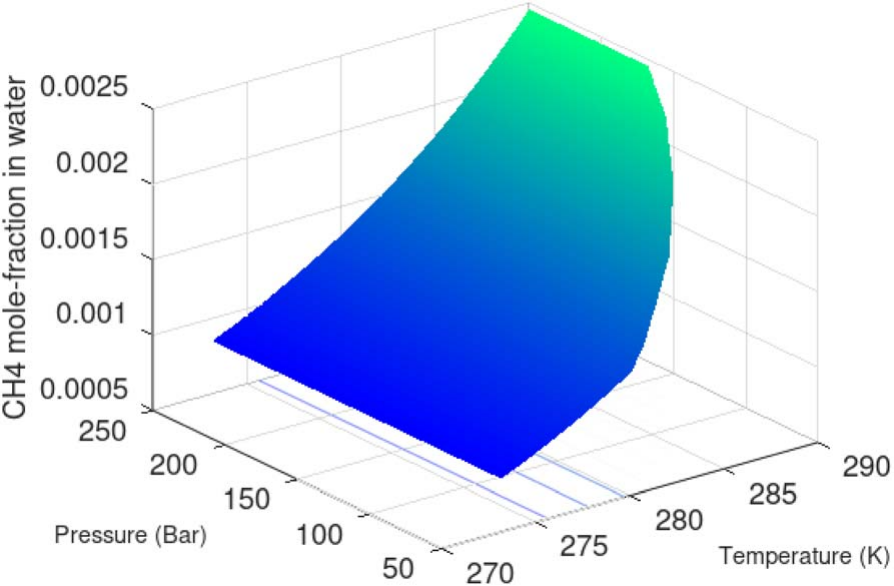
Hydrate stability limits in mole-concentrations of CH_4_ in average seawater (salinity 35) in contact with hydrate.

Superscript w denote aqueous phase and superscript H denotes hydrate. Using equilibrium also for guest molecules in gas and in hydrate we then insert chemical potential for gas in eqn (11) and solve eqn (13) iteratively. In most calculations we choose a range of temperatures and solve eqn (13) for pressures. This is of course the same whether we calculate hydrate stability limit curves, or equilibrium curves. The fundamental difference is, however, that a hydrate stability limit curve is one of several hydrate stability curves and that competing hydrate phase transitions may override the pressure temperature stability limit. This is happening all the time in nature. One example is the dynamics of many offshore hydrates worldwide. Fracture systems that create channels between hydrate filled sediments and seafloor brings in seawater. This seawater contains little or practically no CH_4_ in most cases. CH_4_ hydrate stability limits towards CH_4_ in surrounding seawater are plotted in Fig. 5 below.

A typical situation is that hydrate dissociates towards seawater from top and new hydrate form from upcoming gas. Note that the pressure dependency of this hydrate stability limit is not substantial since it involves two condensed phases. The same type of stability limit is also responsible for hydrate mounds on top of hydrocarbon leakage fluxes worldwide. Upcoming gas forms hydrate due to pressure temperature and dissociates from seafloor side due to limits as plotted in Fig. 5. As a result several levels of biological ecosystems lives on the basis of dissociating hydrate due to CH_4_ concentrations in surrounding seawater lower than limits in Fig. 5.

Also, note that Gibbs free energy for the hydrate is:14
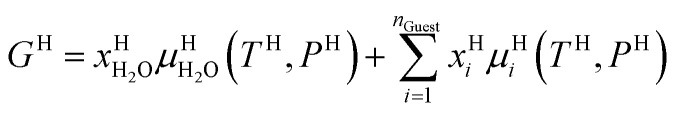


Chemical potential for water and methane along the hydrate stability limits are plotted in Fig. 1b. The change in Gibbs free energy due to the phase transition is given by:15
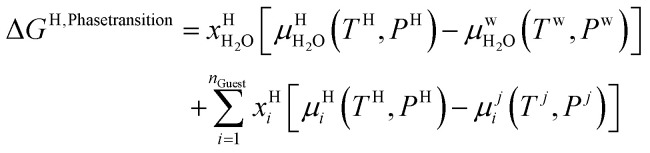


Eqn (15) is zero along the hydrate stability limit in Fig. 1a. The comparison with experimental data in Fig. 1a is therefore a model verification of the chemical potentials for water and guest molecules.

A consistent model for the enthalpy of hydrate formation and dissociation is derived from eqn (15) using the fundamental relationship:^27^16
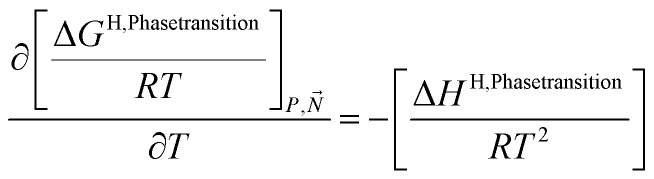


See also ref. 28 Kvamme *et al.* Appendix A for the importance of thermodynamic consistency between calculations of Gibbs free energy and enthalpy. Another advantage of using eqn (15) and (16) is that is it general for hydrate formation from other phases. One example is hydrate formation from dissolved hydrate formers in water. Hydrate nucleation towards mineral surfaces is another example.^1^ Measurements of hydrate phase transition enthalpy is very challenging and critical information, like for instance coordination number, is frequently not available.^30^

## 4. Hydrate phase transition kinetics

In contrast to conventional hydrocarbon systems the hydrate stabilization through water hydrogen bonds imposes nano to meso scale kinetic bottle necks. As will be discussed in more details in Section 7 structured water phases like ice and hydrate gives rice to complex interface between the solid water phase and liquid water. Practically these nano scale interface acts as mass transport barriers. It is therefore not sufficient to supply enough heat to dissociate the hydrate based on hydrate dissociation enthalpy. The level of temperature is also critical. The purpose of this section is to shed more light on these nano scale barriers and possible ways to model these mathematically.

### 4.1. Nano to macro dynamic couplings

Hydrate production dynamic is implicitly coupled on multiple scales. The critical importance of second law, eqn (6) and (7) is on nano to meso scale as directly related to the hydrate phase transitions itself. As discussed before the problem simply boils down to supplying enough heat to break hydrogen bonds and change the entropy from a low (relative) value in hydrate over to higher values in resulting gas and liquid water respectively. The necessary heat supply is macroscopic. The coupling goes through supplied heat flux based on hydrate surface orthonormal to each heat transport direction. If translated over to radial coordinates it is then the heat flux for a certain radial coordinate that faces the surface fraction of hydrates in the pores. Hydrate cannot touch mineral surfaces directly and will always be pore filling.^1–3,21,25,26,28,29^ For a certain radial cross-section area and reasonable simplifications it is even possible to implement a coupled macro to nano model for the entropy dynamics in hydrate reservoir simulators like for instance RetrasoCodeBright (RCB).^34–39^ See also the PhD theses^39–43^ and journal publications included in these theses for more details on RCB and algorithms for handling hydrate phase transitions. To our knowledge RCB is currently the only hydrate reservoir simulator that is feasible for this type of coupling between macro-models for mass- and heat-fluxes, and dynamics hydrate phase transition models which ranges from geometric scales on pore level and down to nano scale on hydrate/liquid water interface. For this reason there are no references to other hydrate reservoir simulators. It is far beyond the scope of this work to detail out a complete model, and associated interface, for RCB within this paper.

**Fig. 6:**
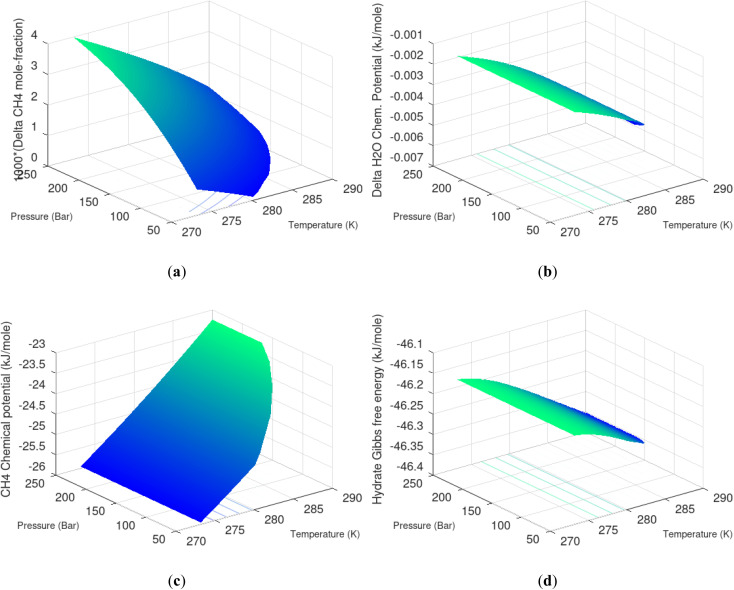
(a) Range of CH_4_ mole-fraction in water for possible homogeneous hydrate formation. Delta is liquid solubility mole-fraction of CH_4_ minus hydrate stability limit mole-fraction of CH_4_. (b) Delta H_2_O is chemical potential difference between hydrate water chemical potential for water in hydrate and in liquid water. (c) Chemical potential for CH_4_ guest molecule from liquid water solution as function of temperature and pressure. (d) Hydrate Gibbs free energy as function of temperature and pressure for homogeneous hydrate formation from liquid water solution of CH_4_.

The thermodynamic control over phase transitions is given Gibbs free energy change across the specific phase transition. At the phase transition limit Gibbs free energy change, eqn (15), is zero and theoretically the hydrate formation rate is the same as the hydrate formation rate is the same and net kinetic phase transition rate is zero. For the heterogeneous hydrate formation on the gas/liquid interface the limits in *P T* projection are given in Fig. 1a. For the homogeneous hydrate formation in Fig. 5 2D projections with either *P* or *T* as fixed variable is obtained by slicing Fig. 5 along either *P* or *T* axis. A fundamental difference between the heterogeneous hydrate formations and the homogeneous formation is that homogeneous hydrate formation can occur in the whole range of concentrations between liquid solubility of guest(s) to hydrate stability limits of guest(s) towards liquid water solution in Fig. 5. Specifically we can use the conditions in Fig. 5 as an example. The difference in liquid solubility mole-fractions of CH_4_ and the mole-fraction for hydrate stability limits in Fig. 5 is plotted in Fig. 6a. The mole-fraction difference is denoted as Delta. The water benefit for the hydrate formation is the difference in chemical potential for water in hydrate and the chemical potential for water in liquid. The sign of Delta is of course opposite in Fig. 6b as compared to Fig. 6a since Fig. 6a is the range of homogeneous hydrate formation possibility while Fig. 6b is the water part of the thermodynamic benefit. Note that every concentration in between liquid solubility of CH_4_ and hydrate stability limit gives rice to a unique hydrate. The reason is that chemical potential for guest molecules vary with concentration in the liquid water solution. This will directly also change hydrate water chemical potential through eqn (11) and (10). Chemical potential for CH_4_ in liquid solution along the concentrations in between solubility and hydrate stability limits in Fig. 6a is plotted in Fig. 6c and Gibbs free energies for the resulting hydrates due to variations in water and guest chemical potentials are plotted in Fig. 6d. The relationship between guest cavity partition functions in eqn (11) and cavity filling fractions is not given explicitly here to save space. The corresponding mole-fractions of water and guests are trivial. See for instance either of the ref. 1, 5 and 29 for details. Every set of concentration, temperature and pressure behind Fig. 6d gives a unique hydrate of specific stability given by Gibbs free energy. This implies that also hydrate dissociation kinetics will be different for each of these hydrate, as discussed more in Section 4.2.

Eqn (9) can be used in two ways relative to the goals of this work. It can be used in a local Gibbs free energy minimization scheme for calculations of phase distributions and associated compositions. Discrete evaluations of specific phase transitions are, however, more relevant for this work. Again we stress the need for consistent calculations of Gibbs free energy, enthalpy and entropy in view of the first term on right hand side of eqn (9). Temperatures of the various phases, as well as external fields imposed by hydrate dissociation stimulation actions, vary dynamically as a function of the impact of hydrate dissociation actions. Eqn (9) can be evaluated for discrete sets of phase transitions. Dissociation through addition of chemicals has so far not been discussed here. For general illustration it is not even needed. It will be discussed in the next Section. See also.^6,44–46^

The two primary terms on right hand side that impose hydrate instability are the first and last term. See Appendix B in ref. 3 Kvamme *et al.* for an example of chemical work.

A critical element in hydrate production is the heat transport needs in the energy balance, and whether the supplied heat is sufficient to supply hydrate dissociation enthalpy.

### 4.2. Hydrate phase transition dynamics

Within the scope of this work Classical Nucleation Theory (CNT) is sufficient for illustration of the most important aspects and also simple enough to be integrated into hydrate reservoir simulators. The two main elements of this theory are (1) formulation of Gibbs free energy change and (2) the hydrate phase transition kinetics. These nano processes are critical for macro aspects of hydrate phase transitions in sediments because they control the onset of phase transition, and in many cases the total dynamics of hydrate phase transitions are dominated by nano scale bottlenecks. Hydrate nucleation is discussed is Section 4.2.1 with focus on macroscopic implications. Classical nucleation theory is discussed is Section 4.4.2.

#### 4.2.1. Hydrate nucleation

Nucleation of a new hydrate involves a competition between favourable clustering towards larger nuclei, and splitting of unstable clusters. This competition contains natural random elements related to the physics of molecular movements in fields of mutual interaction energies. The penalty side of the hydrate nucleation is that the new hydrate nuclei have to push aside molecules in the original phases in order to give space to the nuclei. The balance between the Gibbs free energy benefit of creating the new phase, and the push work penalty, gives rice to a maximum in Gibbs free energy change. After that point Gibbs free energy change for the phase transition will decrease continuously for increasing size of hydrate particles. Using the mathematical condition of a maximum can then fix a critical size parameter and since this point is also a turning point also a second size parameter can be fixed according to the mathematical expression for a turning point. Using both maximum and turning point criteria can for instance parametrize an ellipsoid model for hydrate nuclei^47^ based on the thermodynamics of the phase transition and the penalty term, which is proportional to the interfacial free energy. Using only the maximum criteria can only fix one parameter,^5,28,48^ which is the size of the core at maximum Gibbs free energy. This size is called the critical core radius for a spherical hydrate core, R*.17
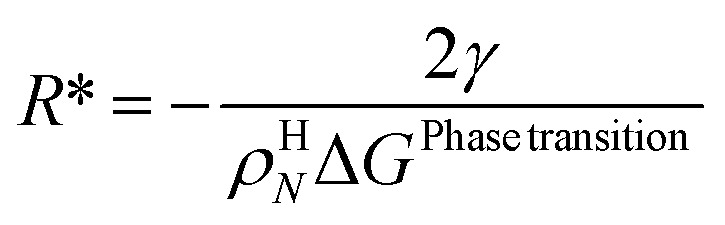


For the simplest possible geometry of a crystal, which is a sphere, with radius *R* we then get:18
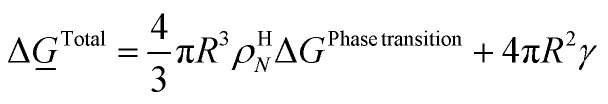


*ρ* ^H^_*N*_ is the molar density of the hydrate core, and in the first term the difference in molar density between hydrate and liquid water is neglected. Δ*G*^Phase transition^ is the molar Gibbs free energy change for the phase transition. *γ* is interface free energy. Frequently, interfacial tension *σ* is used instead of *γ*. Interfacial tension is related to interfacial stress and is one-dimensional, while interface free energy is the work needed to create an interface. The two are related by:19
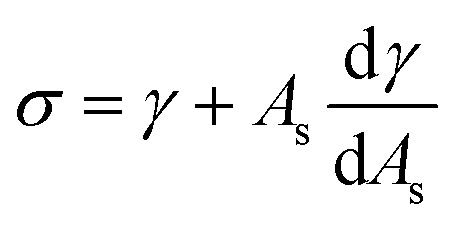


Two things should be stressed. The region beyond critical size is denoted as stable growth, which is technically only true for simple models like CNT. In real systems there can be competition on mass between neighbour growing cores that results in cores of sizes beyond critical size decay because hydrate cores of lower Gibbs free energy will consume mass from hydrate cores of higher Gibbs free energy. These types of effects can be captured in more advanced models like for instance Phase Field Theory (PFT).^8–11,18,19^ Another important thing to keep in mind is that hydrate nucleation, and the subsequent growth phase, are physically well defined processes. Hydrate induction, or onset of massive hydrate growth, on the other hand is a more diffuse definition in the sense that monitoring of hydrate induction time is sensitive to monitoring technology (pressure, laser *etc.*). In open literature, unfortunately, induction times are frequently discussed as nucleation times. Hydrate nucleation is on nano scale in time and space (volume)^5,18,19,21,28,30,47^ while hydrate induction depends on many processes on meso, micro and macro-scale. Hydrate films thinner than 0.1 mm will not be visible to the human eye and experiments based on visual observations, or micro-scale monitoring,^21^ might be misinterpreted as if there is no hydrate present.

**Fig. 7:**
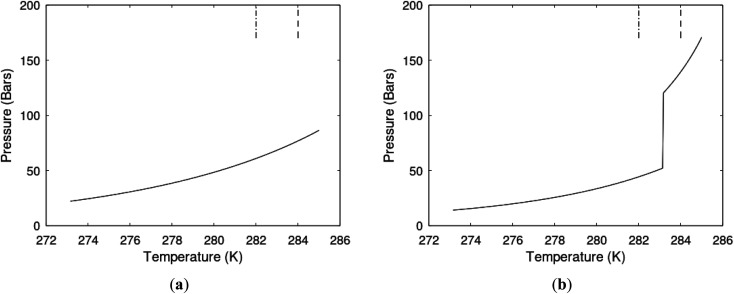
(a) Hydrate stability limits in temperature pressure projection (solid) and two isotherms for varying pressures of supersaturation for CH_4_ hydrate. Dashed is for 282 K and pressures from 170 bar to 200 bar and dash dot is for 284 K and pressures from 170 bar to 200 bar. (b) Hydrate stability limits in temperature pressure projection (solid) and two isotherms for varying pressures of supersaturation for CO_2_ hydrate. Dashed is for 282 K and pressures from 170 bar to 200 bar and dash dot is for 284 K and pressures from 170 bar to 200 bar.

Hydrate production necessarily involves net hydrate dissociation but that does not prevent hydrate reformation to happen. During depressurization the Joule–Thomson effects of released gas cooling bring the system back again into hydrate forming conditions. During the second offshore test in Japan^16^ this was observed as several periods of reduced production before the whole test shut down. Another example is CO_2_/CH_4_ swapping in which the success depends on nucleation and growth of new hydrate from injection gas as heat source for dissociation of *in situ* CH_4_ hydrate. Even during thermal stimulation heat distribution will be non-uniform and might involve regions favourable for hydrate reformation. The use of chemicals for hydrate dissociation, like for instance alcohols, that are solvable in water will be diluted throughout the groundwater, and possibilities of hydrate reformation may result (Fig. 7).

**Fig. 8:**
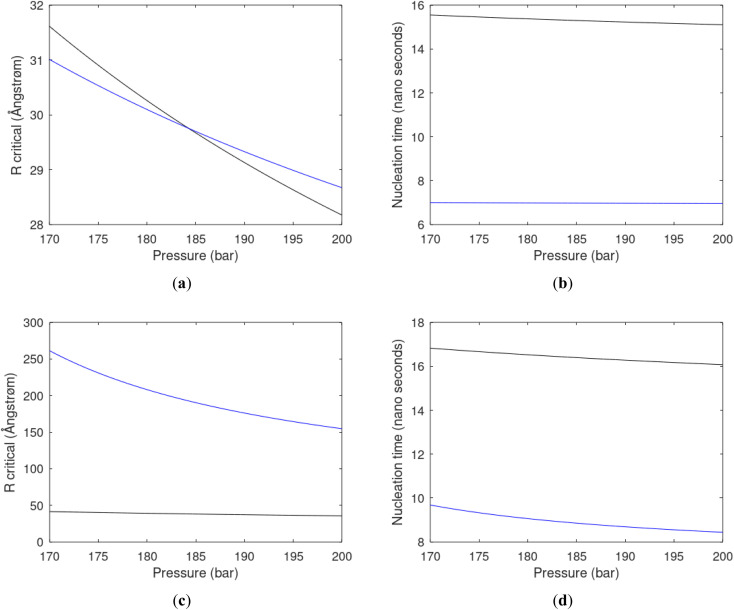
(a) Critical core radius for CH_4_ hydrate (black) and for CO_2_ hydrate (blue) at 282 K and pressures between 170 bar and 200 bar. (b) Nucleation time for CH_4_ hydrate (black) and for CO_2_ hydrate (blue) at 282 K and pressures between 170 bar and 200 bar. (c) Critical core radius for CH_4_ hydrate (black) and for CO_2_ hydrate (blue) at 284 K and pressures between 170 bar and 200 bar. (d) Nucleation time for CH_4_ hydrate (black) and for CO_2_ hydrate (blue) at 284 K and pressures between 170 bar and 200 bar.

As an example here we can look at hydrate nucleation for CH_4_ hydrate and CO_2_ hydrate at conditions relevant for the Black Sea. Two temperatures are fixed – one below the CO_2_ phase transition and one for a temperature above the phase transition point (Fig. 8).

**Fig. 9:**
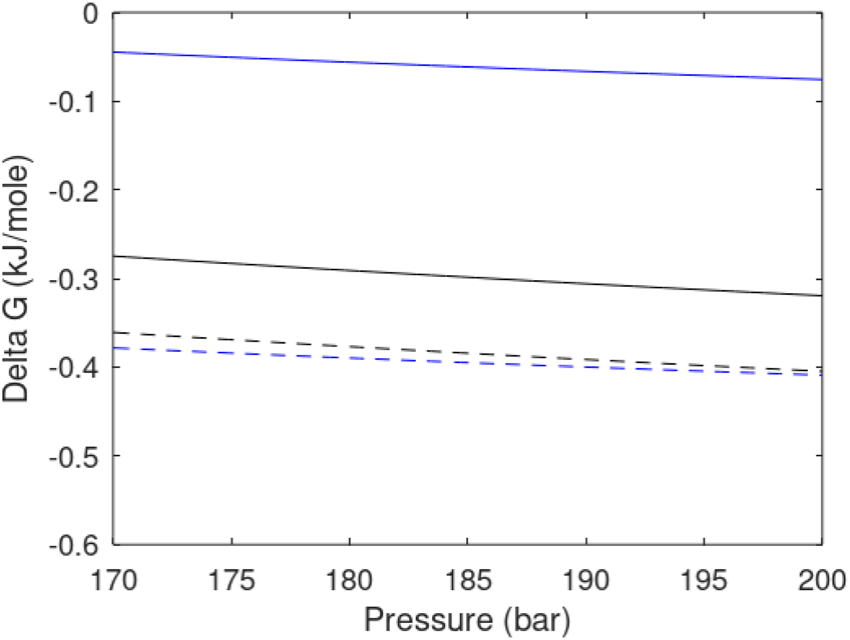
Gibbs free energy changes for hydrate formation at 282 K (dashed) and 284 K (solid). Blue is for CO_2_ hydrate formation and black is for CH_4_ hydrate formation.

Free energy changes for hydrate formation are plotted in Fig. 9. When both CH_4_ and CO_2_ are in gas region the critical radii are not very different. The CO_2_ phase transition for CO_2_ changes that and critical radius for CO_2_ hydrate is larger than for CH_4_ hydrate. CO_2_ hydrate is, however, a substantially more stable hydrate than CH_4_ hydrate and nucleates faster at both temperatures. Gibbs free energy change for the formation of CO_2_ hydrate at 284 K is significantly smaller in absolute value than the corresponding value at 282 K because the change in density (and molecular attractions) for guest phase to hydrate is smaller.

**Fig. 10:**
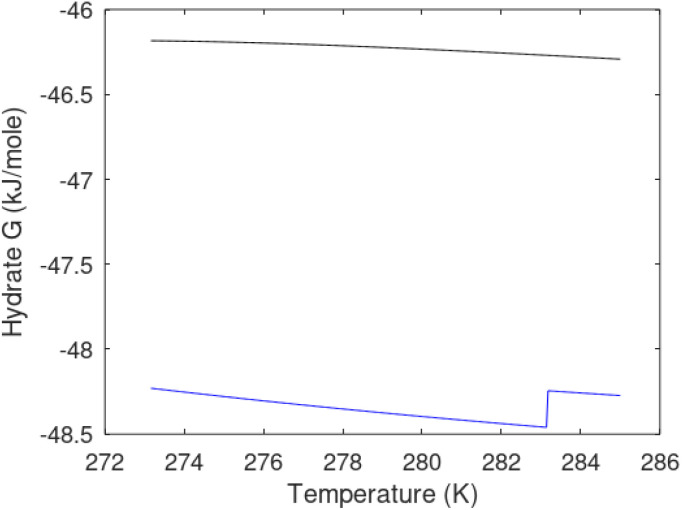
Gibbs free energy for hydrates formed from CO_2_ (blue) and from CH_4_ (black).

It is also important to keep in mind that for conditions in which both CH_4_ hydrate and CO_2_ hydrate can exists the CO_2_ hydrate is always more stable (lower Gibbs free energy) than CH_4_ hydrate as illustrated in Fig. 10 below.

The practical implications of this is that CH_4_ hydrate will nucleate instantly on a macroscale in time (seconds and up) if the thermodynamic conditions are favourable. The paradox is that the Joule–Thomson cooling effect per pore volume will be proportional to the volume fraction gas in the pores. Turbulence, and associated large gas/water contact areas, will further stimulate to generation of large numbers of hydrate nuclei, as well as fast hydrate growth and accumulation of hydrate cores. In CO_2_/CH_4_ swapping method CO_2_ hydrate nucleation will totally dominate relative to possible reformation of CH_4_ hydrate. These are just two practical examples of the need to evaluate the nano scale aspects of hydrate phase transition dynamics.

**Fig. 11:**
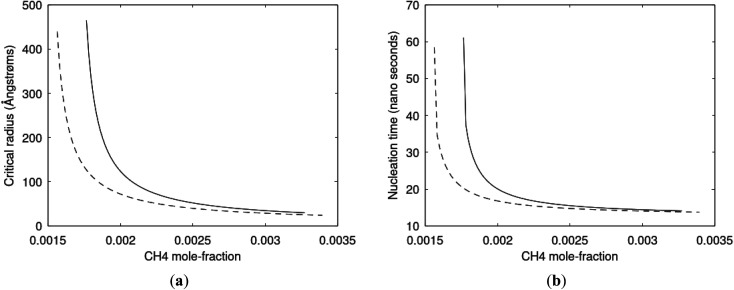
(a) Critical radii for hydrate formed homogeneously from dissolved CH_4_ in water at 282 K and 170 bar (dashed) and 284 K and 170 bar (solid) as function of CH_4_ concentration in water between solubility concentration and minimum hydrate stability concentration. Minimum mole-fraction CH_4_ in outside liquid water for hydrate stability is 1.49 × 10^−3^ at 282 K and 170 bar and 1.68 × 10^−3^ at 284 K and 170 bar. (b) Nucleation time for homogeneous hydrate formation from CH_4_ dissolved in water. Conditions and notations as in (a).

**Fig. 12:**
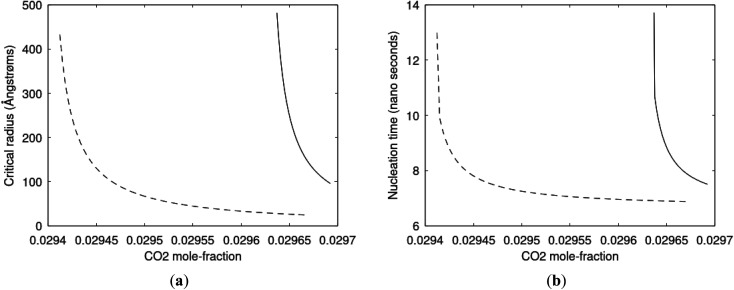
(a) Critical radii for hydrate formed homogeneously from dissolved CO_2_ in water at 282 K and 170 bar (dashed) and 284 K and 170 bar (solid) as function of CO_2_ concentration in water between solubility concentration and minimum hydrate stability concentration. Minimum mole-fraction CO_2_ in outside liquid water for hydrate stability is 2.94 × 10^−2^ at 282 K and 170 bar and 2.96 × 10^−2^ at 284 K and 170 bar. (b) Nucleation time for homogeneous hydrate formation from CO_2_ dissolved in water. Conditions and notations as in (a).

**Fig. 13:**
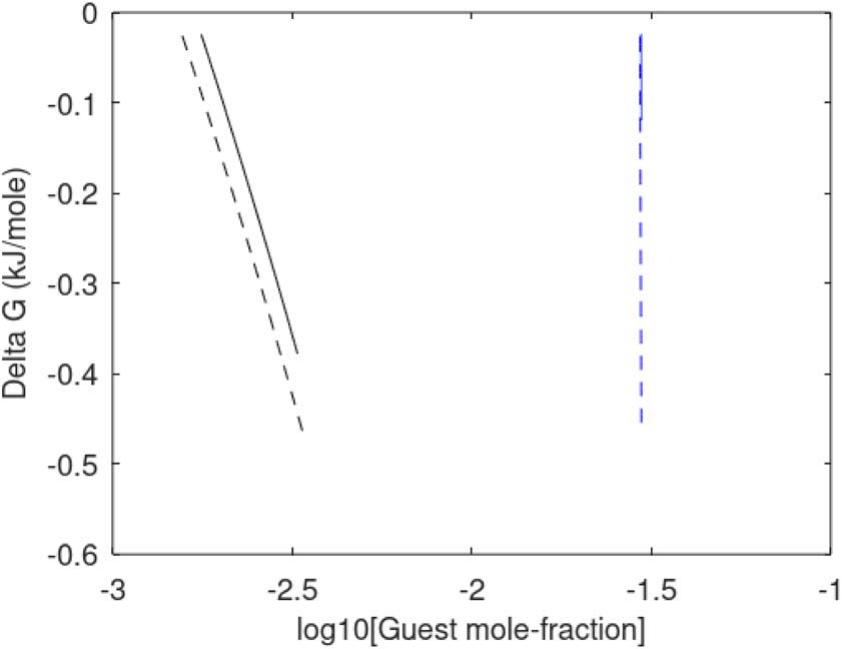
Gibbs free energy change for hydrate formation as function of concentration of guest molecules in water. Black is CH_4_ and blue is CO_2_. Dashed is for 282 K and 170 bar and solid is for 282 K and 170 bar.

Since also heterogeneous hydrate nucleation is dominated by the liquid water side of the interface homogeneous hydrate nucleation from guest molecules dissolved in water. Critical radius and nucleation times for representative conditions comparable to the heterogeneous case are plotted in Fig. 11 for CH_4_ and in Fig. 12 for CO_2_. Gibbs free energy changes for hydrate formation are plotted in Fig. 13.

As expected the critical radii are very large for liquid concentrations of guest close to limit of hydrate stability concentrations. Corresponding nucleation times are relatively high but still on nano scale and instant relative to macro time scales of seconds and longer times. Closer to solubility concentrations the critical core radii are slightly larger than heterogeneous hydrate formation. For CO_2_ there is a distinct change from 282 K to 284 K. In a turbulent situation during hydrate dissociation homogeneous nucleation from supersaturated water, as well as heterogeneous hydrate nucleation in liquid water side of interface will represent two end points of a chain of hydrate nucleation situations in which the homogeneous case is the asymptotic case of homogeneously dissolved guest in water.

Hydrate dissociation does not involve the creation of a totally new phase since hydrate facing another phase will result in a development of an interface. For hydrate in contact with water we have already discussed the interface as a nano scale dynamically reproducing interface due to difference in distributions of water partial charges. Hydrate facing gas also need to restructure in order to optimize surface entropy since fixed location hydrate water partial charges facing non-polar guest phase is in entropy penalty. Also in this case a hydrate/gas interface of structured water will develop. For hydrate dissociation there are no direct penalty terms as that in eqn (18) for nucleation of hydrate cores. What is common to hydrate nucleation and growth, and dissociation is the hydrogen bonded interface, which can be a macroscopic bottle-neck.

#### 4.2.2. Classical Nucleation Theory

Classical Nucleation Theory (CNT) can be expressed by:

20 *J* = *J*_0_ e^−*β*Δ*G*^Total^^

where *J*_0_ is the mass transport flux supplying building blocks for the hydrate growth. For the heterogeneous hydrate phase formation it will be the supply of methane to the interface growth. For homogeneous hydrate formation it will be the diffusion rate for dissolved methane to hydrate crystal growth from aqueous solution. The original CNT is limited by a classical pre-factor *J*_0_ for single pure component transport. It was utilized by Kvamme^48,49^ in Multicomponent Diffuse Interface Theory (MDIT). Looking forward, we need an approach that can be developed also for multicomponent mixtures. This is needed since hydrate will not form from “bulk” hydrate formers but rather from hydrate formers adsorbed on water interface, and correspondingly a super-saturated water interface.

Eqn (21) below, with parameters in Table 1, is the equation applied for Fig. 3. At this stage, eqn (21) is considered as semi-empirical since the samplings of diffusivities from MD are fairly uncertain. Parameters in Table 1 are the same for CH_4_ and CO_2_ since the diffusivity is controlled by hydrogen bonded water.21
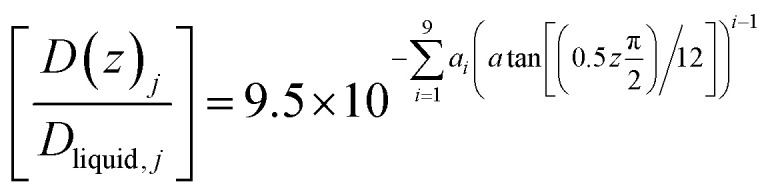


**Table 1:** Parameters for eqn (21)

*I*	Parameter	*i*	Parameter	*I*	Parameter
1	0.979242	4	171.673	7	−9649.96
2	15.5427	5	6.76975	8	14 779.7
3	−88.5112	6	1939.55	9	−7496.15

The time for a methane molecule to cross the interface can be found by integrations of the second order Fick's law:22
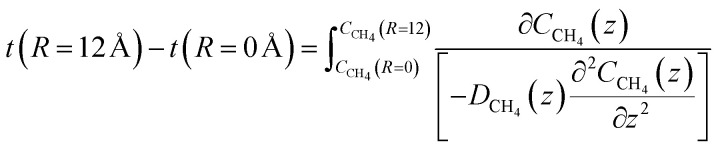


The liquid water/hydrate interface is constantly reproducing itself on nano-scales in time. The mass transport is dynamically limited by the very low diffusivity close to the hydrate surface. A fair approximation is therefore:23
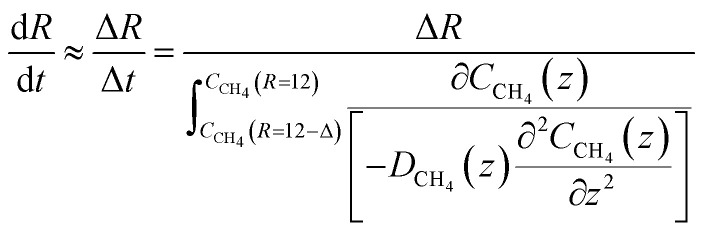


For hydrate growth beyond interface thickness, eqn (23) is used as a constant value for film thickness growth. The corresponding mass transport dynamics in CNT, *J*_0_, is then given by:24
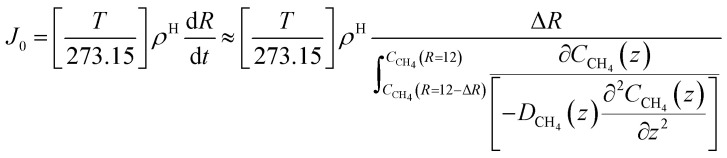


*ρ* ^H^ is the molar density of the hydrate. This is trivial to calculate from the volume of the hydrate (unit cell dimension) and filling of the cavities. Diffusivities based on auto velocity correlation samplings scale proportionally to square velocities. The relationship between kinetic energy and temperature per degree of freedom for movement gives the temperature scaling relative to the MD simulations that were conducted at 273.15 K. Within other uncertainties this scaling is not very significant.

The implicit coupling to heat transport is then

25 *Q̇* ∼ Δ*H̲*^Total^

In which the enthalpy change is given eqn (16) and Total is the same as Phase transition for hydrate dissociation while the penalty term in eqn (18) applies to the hydrate nucleation stage during hydrate formation. This term rapidly fades to almost zero, relative to the phase transition Gibbs free energy benefit term, for increasing hydrate core sizes beyond critical radius size. Except for calculations of nucleation sizes, as illustrated in the previous section it can be omitted in the hydrate growth region. Two various ways that heat can be transported are discussed in Section 2, eqn (3) and (4).

As an example we may consider CH_4_ hydrate dissociation towards incoming water from seafloor. Example is artificial but condition partly representative for Black Sea. An *in situ* CH_4_ hydrate at 284 K and 170 bar faces incoming water with 282 K and 170 bar. The corresponding thermodynamic control farctor, the exponent with Gibbs free energy change in eqn (20) is 1.39. The diffusivity coefficient for liquid water side of interface is assumed to be 1 × 10^−8^ m^2^ s^−1^, hydrate side interface diffusivity coefficient is 1 × 10^−12^ m^2^ s^−1^. The calculated molecular flux of CH_4_ from hydrate is calculated to 39 975 mol Å^−2^·s^−1^, which corresponds to 6.638 mol m^−2^ s^−1^ or 0.1062 kg m^−2^ s^−1^.

## 5. Black sea hydrates distribution, saturations, and volumes

The unique conditions of the Black Sea for gas hydrate existence made the basin the most hopeful for hydrate exploitation in Europe. The main gas hydrate deposits (GHDs) of the Black Sea are located in the Danube Fan in the northwestern part of the basin. Published estimations of hydrate saturations (*S*_h_) are used for raw hydrate volumes (*V*_h_) determinations.

### 5.1. Black sea basin unique conditions for hydrates existence

**Fig. 14:**
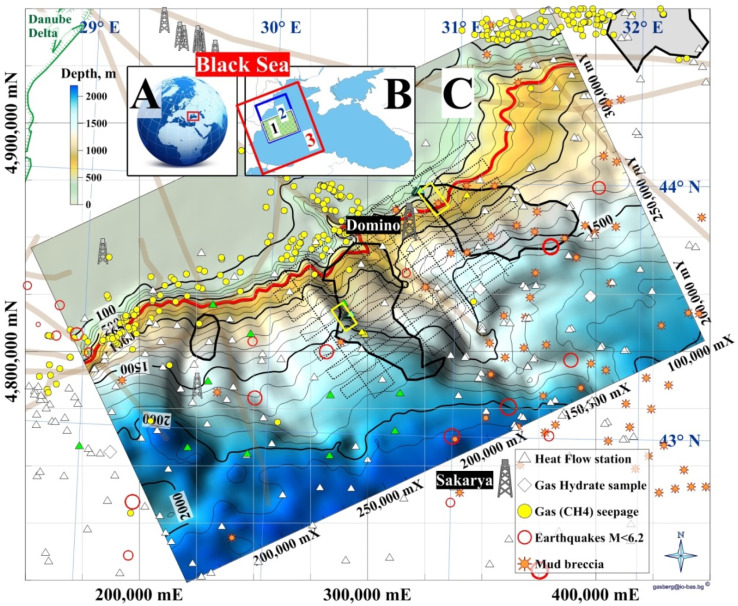
Study areas. (A) Black Sea; (B) (1) area presented in (C); (2) a model area for (1); (3) Western Black Sea basin analysis model;^53^ (C) Bathymetry and GHDs in the Danube paleodelta (cruise MSM34-35, SUGAR III project). (Legend) 4 dimed polygons: from left to right 2 Bulgarian, Romanian and Ukrainian GHDs/BSR areas; red isobath 650 m: MHSZ lateral boundary; 2 yellow rectangles: SUGAR III detail 3D seismic & CSEM polygons; dotted lines: MSM34 2D seismic; thick grey lines: faults; Black Sea biggest conventional natural gas fields: Sakarya (405 bcm; Turkey^54^) and Domino (42–84 bcm; Romania^55^).

The focus area is the northernmost part of the Bulgarian Exclusive Economic Zone (BEEZ) of the Black Sea where seismic records with registered bottom simulating reflectors (BSRs)^50,51^ show on the continental slope a significant GHD with an area of ∼1300 km^2^ (65 × 20 km) at depths 300–400 mbsf (meters below seafloor). The area is part of the deep-water Danube Fan. GHD with almost the same area exists in the Romanian Exclusive Economic Zone (REEZ) northern from the Viteaz Canyon^51,52^ (Fig. 14).

The area is the transition zone between the Moesian Platform in the west, the Scythian Platform in the north, and the Western Black Sea basin in the south-east.^56^ The East Moesian Trough and the Polshkov Ridge are located in the western part of area 1 in Fig. 14. Two faults starting from the area of the present Danube delta reach the northeastern and the southwestern corner of the MSM34 seismic work area (Fig. 14). The axis of the area is the marine Peceneaga-Camena fault, which follows the main modern levee channel of the Viteaz Canyon.

The adjacent onshore areas are mainly drained from the Danube. Sedimentation in the large deep-sea fan complexes is controlled by climate and sea level changes.^57^ The sedimentary architecture is determined by the turbidity system of the Danube deep-sea fan. This is the largest mud-rich fan in the basin, composed of stacked channel-levee systems.^56^ Channel-levee systems are lenticular sedimentary units with coarse-grained sediments at the channel axis, and fine-grained, alternations of sand and mud in the lateral levees.^58^

The presence of GHs was inferred from seismic records with bottom simulating reflectors (BSRs), which marked the base of the GHSZ.^51,52^ BSRs were identified in the youngest buried levee-channel systems of the Danube northern and southern from the Viteaz Canyon.^51^ The BSR area in the BEEZ lies on the continental slope at water depths 750–1830 m. Unusual quintuple BSR was reported in the area^59^ which focused the further detail 3D seismic and CSEM investigations (yellow frame in Fig. 14C). The south GHD difference from the north GHD is that it is well sealed and gas seepages and enhanced methane values in the seafloor sediments were not determined.^52^ GH reservoirs with massive sand deposits are expected within the levee-channel systems,^52^ which makes the area promising for the first tests for GH exploitation in EU.^52^

After the discovery of the wide areas with BSRs (EC project ASSEMBLAGE) and the first systematic 2D multichannel seismic (Institute of Marine Sciences and Technology, Izmir, Turkey for the German project SUGAR III) all interpretation efforts were concentrated in the detail polygons with sizes ∼15 × 5 km. That's why all cited estimations are about the area of the detailed polygon in the BEEZ.

The unique conditions of the Black Sea GHs are determined by the lower salinity and the higher temperature of the near bottom waters in comparison with these in the oceans – 35 PSU and 0–3 °C. Their last significant variations are due to climate and sea level changes after the Last Glacial Maximum (LGM).^57^ Due to the low salinity of the Black Sea waters of ∼22 PSU, the more dense winter waters formed mostly in the area of the northwestern shelf, submerse only to depths of ∼150 m. As a result, in the Black Sea waters under 150 m the O_2_ is replaced with H_2_S and less CH_4_ and forms the anoxic zone (∼90% of the water body). The temperature of the waters under 500 m (the depth of insulation for the Black Sea) is almost constant at 9.1 °C according to more than a century of measurements.^60^

The heat flow of the central part of the western Black Sea is <20 mW m^−2^, which is below the heat flow of the old oceanic basins. These lowest values are due to recent sedimentation and the result is a non-equilibrium temperature field. The northwestern margins' heat flow values are typical for the continental crust (50–70 mW m^−2^) but *in situ* heat flow measurements in the detail polygon show values of 30–50 mW m^−2^ and an average geothermal gradient of 30 mK m^−1^.

The salinity in sediments decreases from 22 PSU on the seafloor to 2–3 PSU at a depth of 40 mbsf and down is almost constant.^59^

Hydrates in the Danube Fan are of microbial origin (methane *δ*^13^C −84‰ to −70‰) and concentrations of 99.1–99.9%.^65^ Drilling with MARUM MeBo200 in the northwestern part of the GHD in the REEZ showed little amounts of thermogenic gases and methane was the dominant gas in sediments of the upper ∼150 mbsf.^66^

Concluding, the Black Sea conditions are favorable for large GHDs formation. Only the higher seafloor temperature is with some negative effect – a shallower GHSZ but the trapping of the hydrate former gases in a thinner GHSZ could create a higher hydrate saturation.

### 5.2. GHD in the BEEZ: hydrate saturations

Two main layers with GHs are registered from seismic and CSEM – shallow at depths 100–150 mbsf and deep/main with a base at the BSR at depths 300–400 mbsf. According to seismic the shallow layer is with lower saturation opposite to the CSEM results (Table 2).

**Table 2:** GH saturation (*S*_h_) of the sediments of the GHD in the BEEZ was determined from seismic and CSEM in the polygon for a detailed 3D study at a water depth of ∼1500 m. The maximum value in the column “Sediments depths” shows the depth of the main BSR (BSR1)

Geophysical method	GH saturation average, %	GH saturation max, %	Sediments depths, Mbsf	Source
Seismic	12	25	80–150	56
	20		300–400	
CSEM	20–30	60	100–150	61
	10	30	300–400	
Heat flow	38	73	230–330	62

The deeper GH-saturated horizon is registered with BSRs almost in the whole area.^59^ An exception is the central part of the GHD area where the BSR is horizontal as the main lithostratigraphic boundaries and cannot be recognized.^59^ The upper GH saturated horizon is registered on a few records and usually with lengths of first kilometers. According to CSEM some parts of the upper horizon could be with average GH saturation of >30%. Heat flow results are from the model of the deeper/main horizon and show higher average and maximum GH saturations. The heat flow method is with a higher ratio of signal/noise than seismic and CSEM.^62^ Its results are comparable with those from the basin analysis.^53^

**Fig. 15:**
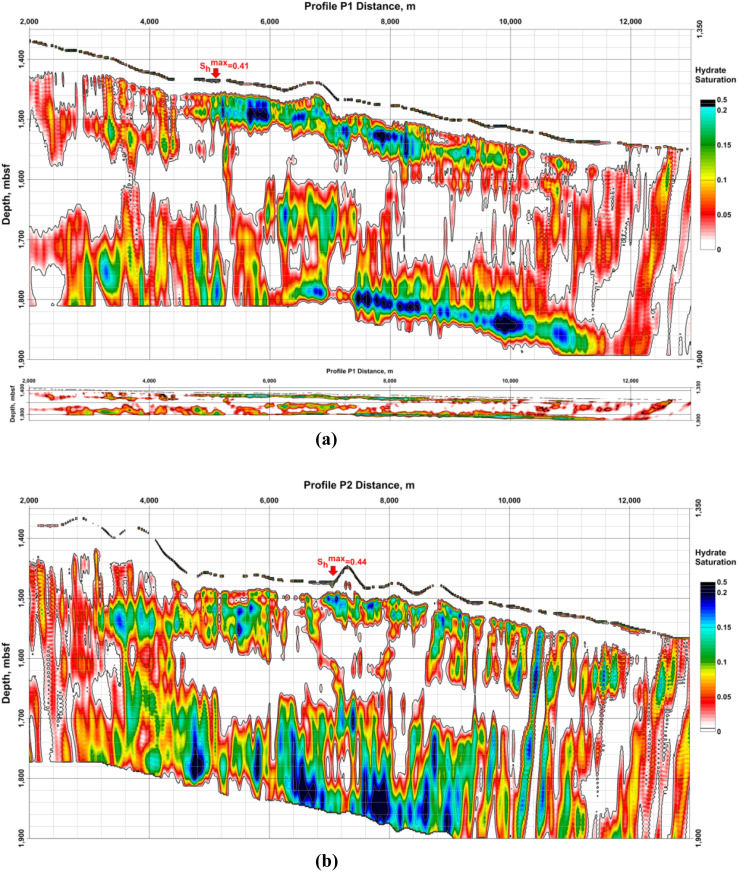
Hydrate saturation (*S*_h_) from Gassner *et al.*:^58^ (a) profile P1; up – with vertical exaggeration; down – with the same horizontal and vertical scale; (b) profile P2. Both model grid sizes are 7,200 × 1,500 and cover 14.4 km in length and 3 km depth under the sea level. Horizontal and vertical steps are equal to 2 m. Profile P1 is parallel to P2 and the distance between them is 1 km. *S*_h_ represents the hydrate saturation of the pore space. The number of the OBS stations on every profile is 5 with 1 km spacing between them. The maximum values on the profiles P1 and P2 of *S*_h_ > 0.4 are in the uppermost sediments (near-bottom). The vertical exaggeration is ×10.

Data provided by Dr Laura Gassner^58^ present hydrate saturation from the detailed polygon of the GHD in the BEEZ in Fig. 15. Results from acoustic full-waveform inversion (FWI) of ocean bottom seismographs' (OBS) data show that hydrates are connected to higher P- and S-wave velocities while the free gas under the BSRs reduces P-velocities (*v*_P_) only.^58^

A limitation of the method is the assumption that hydrates could exist only above the BSR and gas – only below the BSR.^58^ For the sediment volumes where hydrates and gas coexist model results will show decreased values for both – hydrate and gas saturations because the assumption in these cases decreases P-wave velocities above the BSR and increases them bellow the BSR. Information about the FWI.^58^ Other examples, suggesting that the results for hydrate saturation are decreased are that the Gaussian smoothing applied to obtain starting models decreased interpreted signal anomalies; *v*_P_ increase above the BSR and *v*_P_/*v*_S_ ratio decrease are limited; assumed no effect of the hydrate and gas on the density model, *etc.* Such reasons could explain the absence of gas in the model results under the registered BSR on profile P2.^58^

Calculated hydrate and gas saturations are up to 30% and 1.2%.^58^ The saturations depend on the assumed sediment composition and porosity. A better estimation needs drilling and direct parameter measurements.

Imagination with so high resolution is not possible to receive with drilling and therefore is useful in showing the challenges for hydrate production technologies due to the jumps in saturation values.

### 5.3. GHD in the BEEZ: hydrate volumes

The GHD in the BEEZ is designated as promising in Europe for implementation of technologies for recovery of methane from hydrates^52^ because of expected significant hydrate reserves in sand collectors at distance ∼200 km from the main Bulgarian port Varna (the closest to the GHD is the Bulgarian port Shabla – 135 km; the main Romanian port Constanta is at 150 km). For a raw calculation of hydrate reserves of a GHD are needed estimations of the next parameters:

• average part of the GHSZ sediment volume with hydrates;

• average hydrate saturation;

• average porosity (relative volume of the sediment pore space);

• the average thickness of sediments in the GHSZ (the BSR depth under the seabed);

• lateral area of the GHD.

#### 5.3.1. Average parts of the GHSZ sediment volume with hydrates

Results shown in Fig. 15 (ref. 58) determine the parts of sediments between the seabed and the BSR with hydrate saturations 0% ≥ 30% (Table 3).

**Table 3:** Parts of the areas (*S*) for profiles P1 and P2 relative to the total area of their sediments between the seafloor and the BSR, their average hydrate saturations (*S*_h_), and their average values (average) for sediments with *S*_h_ > (0–30)% and *S*_h_ step 5%. The total area for profiles P1 and P2 in Fig. 15 is 6.050 × 10^6^ m^2^. For profile P1 the area of sediments between the seafloor and the BSR is 3.94 × 10^6^ m^2^ and for profile P2 – 4.28 × 10^6^ m^2^

Profile	*S*, %	*S*, %	*S*, %	*S*, %	*S*, %	*S*, %	*S*, %
	*S*_h_, %	*S*_h_, %	*S*_h_, %	*S*_h_, %	*S*_h_, %	*S*_h_, %	*S*_h_, %
	*S*_h_ > 0%	*S*_h_ > 5%	*S*_h_ > 10%	*S*_h_ > 15%	*S*_h_ > 20%	*S*_h_ > 25%	*S*_h_ > 30%
P1	82.0	29.2	10.7	3.2	0.51	0.0085	0.0012
	4.7	9.6	13.8	17.7	21.6	27.4	33.0
P2	81.4	42.5	17.4	4.9	1.02	0.0536	0.0019
	6.2	9.9	13.8	18.0	21.9	26.1	35.7
Average	81.7	35.8	14.0	4.0	0.76	0.0310	0.0015
	5.4	9.8	13.8	17.8	21.8	26.8	34.4

For the GHD raw total hydrate reserve estimation, we use the values in Table 3 for the average part of the GHD sediment volume with hydrates of 81.7% and its average hydrate saturation of 5.4%.

#### 5.3.2. Average porosity

MeBo200 drilling from 3 boreholes in the GHD area in the REEZ to the depths up to 147.3 mbsf at water depths 765–860 m shows an average depth profile of porosity with near-seafloor values of up to ∼0.9 which steeply decline to ∼0.5 at ∼20 mbsf and is with small variations in 0.4–0.5 at depths ∼40–150 mbsf.^63^ The average porosity was assumed to equal 0.45 accounting for the general trend of porosity decrease with the depth increase and that the BSR1 depths in the Danube area are >450 mbsf.^64^ Although drilling was set to characterize hydrates in channel-levee sediments with favorable conditions for higher hydrate saturation all data suggest no gas hydrate is present at drill sites.^63^

#### 5.3.3. Average GHD BSR depth

The average depth of the BSR has assumed 350 mbsf based on the published results:

• the average GHSZ depth of the GHD in the BEEZ is ∼350 mbsf;^60^

• the shallowest (main) BSR occurs in the area at depths 320–380 mbsf^59^ (average 350 mbsf);

• at a water depth of 765 m (close to the shallowest water depth of the GHD in the BEEZ) the BSR depth is 144 mbsf^63^ and only the maximum BSR1 depth in the Danube area is > 450 mbsf.^64^

#### 5.3.4. Lateral area of the GHD

The lateral area of the GHD in the BEEZ is determined from the polygon – outer boundary of the GHD^59^ in the map in Fig. 14 to 1430 km^2^.

#### 5.3.5. Raw hydrate reserve estimation of the GHD in the BEEZ

The raw pure hydrate estimation of the GHD in the BEEZ using the above estimations and assumptions shows hydrate reserves of ∼10.0 bcm (1430 × 0.35 × 0.817 × 0.45 × 0.054 km^3^; 1 km^3^ = 1 bcm) and methane reserves of ∼1550 bcm (in average 155 STP volumes methane in 1 volume natural methane hydrate). To receive this quantity of methane is needed to process ∼80% of the sediment volume, but the main quantity of hydrate of ∼8.0 bcm is in ∼36% of the sediment volume with average hydrate saturation of ∼10.0% (Table 3). The yearly consumption of natural gas in Bulgaria of ∼3 bcm (2020) is equal to the average gas reserves in the area of ∼3 km^2^ of the GHD in the BEEZ.

This estimation is probably 2 times lower than this from CSEM and 3 times from the heat flow approach (Table 2). Therefore, the actual gas quantity is likely to be >3000 bcm. A conventional gas field with the closest gas reserves is the 5th largest in the world (Shtokman, Russia, 3100 bcm). The reserves of the GHD in the BEEZ are 2 times more compared with the cited down largest Norwegian gas field Troll with originally recoverable reserves of 1437 bcm (area ∼50 × 20 km; remaining reserves ∼685 bcm). About 277 production wells, 599 sidetracks and more than two million reservoir metres drilled on Troll produce yearly ∼40 bcm gas.

The presented results demonstrate the main obstacles to model estimations and future production tests. Final results will be available only after the realization of a drilling program.

## 6. Dominating thermodynamic processes for various methods to release natural gas from hydrates

It can be useful as an initial qualitative overview to look at which thermodynamic contributions are affected by different sets of actions intended to dissociate hydrate (Table 4).

**Table 4:** Qualitative overview of active contributions towards hydrate dissociation. Numbers are also qualitative indications that ranges from 0 as “not effective” to 10 as effective

	(1) Law			Combined law			(2) Law
	Heat	Shaft *W*	Chem. *W*	Heat *S*	Shaft *W*	Chem. *W*	*T*
P-reduction	1–3	0	1	1–3	0	1	0
Thermal	8–10	0	5	8–10	0	5	10
Chemical	0	0	10	0	0	10	10
CO_2_	5–10	1–5	3–6	5–10	1–5	3–6	10

Shaft work is the second term on the right-hand side of (1) law and stems from Legendre transform from energy to enthalpy, in which the internal “push work” is subtracted. Shaft work may be denoted as “exportable work”. Practically it contributes to Joule–Thomson cooling by gas expansion. It also occurs in Gibbs free energy, which is critical for the evaluation of the most likely phase distributions.

Heat is the first term on the right-hand side of the first law while heat *S* is the impact of the second law on Gibbs free energy. In a simplified way, Gibbs free energy can be interpreted as “available energy” since it is the energy level for flowing systems (enthalpy) minus the energy of irreversibility. Thermodynamically irreversibility is frequently named friction – in a wider interpretation of friction than simple mechanical friction.

Chemical work is the net molecular work involved in transferring molecules from one phase over to another phase. The net energy part of chemical work is the change in interaction energies with other molecules while the entropy part of it is the rearrangements of the two phases after the transfer of molecules. See also Appendix B in ref. 3 Kvamme *et al.* for some examples of possible models for chemical work. A numerical example is given in the last paragraph of Section 4.

*T* in the column for (2) law has a more extended meaning than just temperature. For thermal stimulation and injection of CO_2,_ it is the real temperature level. It is simply an indication that the level of temperature has to be high enough to break hydrogen bonds and provide the necessary entropy increase from hydrate over to proportional amounts of gas and liquid water. In the extended meaning of adding chemicals for breaking hydrogen bonds it simply means the possibility of achieving that goal.

Table 4 is purely qualitative and just an overview as basis for more quantitative calculations, and for possible proposals that can improve the actual method.

Every hydrate reservoir is different and ranges from deposits with no free gas and high hydrate saturation, *via* no free gas and low hydrate saturation to all variations of sediments containing gas/liquid water and hydrate.

On top of that there are the variations in sediment distributions that can range from hydrates on top of conventional gas like for instance Messayokha in Siberia.^65^ Since 1970 it is estimated that about half of the produced gas from that is from dissociation of hydrate. From the time-lines for pressure changes^65^ it appears that the dissociation of hydrate has been slow. But it is a huge conventional gas reservoir. Some of the many wells are periodically closed and stimulated by methanol to remove hydrate plugging. To repeat – Table 1 is merely a list of challenges related to each of 4 different categories for stimulation and promotion of hydrate dissociation. Some associated calculations are presented in Section 7.

## 7. Advantages and limitations of methods to release natural gas from hydrates

The variety of hydrate deposits around the world is huge. It will be impossible to address the whole range of differences in gas/water/hydrate distributions in hydrate filled sediments, sediment characteristics and all other key parameters that dictate the dynamic state of the hydrate sections. And on top of that the impact of all surrounding sediments, including fracture systems that transports hydrocarbons in to hydrate filled sediments as well as fracture systems that brings in seawater and causes hydrate dissociation. Pin-pointing some challenges and suggesting possible improvements is a main motivation for this section.

Several hydrate reservoir simulators are available in the open literature. Some are academic and open while others are commercial and frequently lack details on algorithms and built in parameters. A general challenge is the numerical complexity, which frequently limit the rigor of the models for phase transitions. One example is hydrate formation, and hydrate dissociation, which are frequently just an “on/off” which in only temperature and pressure for a local grid cell in a simulation. For CH_4_ hydrate, as one example, it is simply a question of whether conditions are inside or outside the hydrate stability limits in Fig. 1a, regardless of other competing hydrate phase transitions and associated dynamics in a non-equilibrium system. Frequently oversimplified empirical kinetic models, like the one due to Kim *et al.*^66^ are used. This correlation is based on controlled laboratory experiments in a cell with different levels of stirring and very different from conditions in natural sediment. It is not even based on the correct mechanism since all experiences from pilot plant studies (see earlier discussion in introduction) as well as theoretical considerations points on heat supply limitations for the pressure reduction method. The only way forward is a kinetic model that contains consistent thermodynamics and realistic mass transport barriers, as discussed in Section 4. See also Kvamme and Clarke^5^ for a review of old models and discussion of some possible pathways forward. RetrasoCodeBright^33–43^ is in many senses the only platform for hydrate reservoir simulations that can include competing hydrate phase transitions because it has a built in free energy minimization module. The numerical challenges are still substantial for some problems.

Within the focus of this work it is more important to evaluate the various thermodynamic contributions to hydrate phase transitions. Evaluation of specific models for the various contributions to the first law of thermodynamics (enthalpy) for energy balances, the combined law (Gibbs free energy) for phase stability and second law (entropy) for level of temperature needed to provide necessary entry increase from solid hydrate to higher level as free gas and liquid water entropies. Relative impact of the various contributions in eqn (1)–(9) and nano scale bottlenecks related to hydrogen bonds in hydrate and hydrate/liquid water interface is the main goal. Simple models intended to at least reflect potential challenges of the different methods to release natural gas from hydrates are therefore a good starting point.

Some challenges related to pressure reduction are discussed in Section 7.1 and also illustrated with simple calculations based on literature values for transport properties as systemized by Kvamme *et al.*^7^ There are many ways to add heat and Section 7.2 is mainly devoted to qualitative evaluation of the economic feasibility. Adding methanol or various chemical that breaks hydrogen bonds can be efficient locally. Salts are corrosive but also efficient. Section 7.3 is limited to some thermodynamic calculations and associated cost estimates. Injection of CO_2_ with additives, as discussed in Section 7.4, has some unique features like combined CO_2_ storage and energy production. Since new hydrate from injection gas forms inside the pores between sediments and *in situ* CH_4_ hydrate it is efficient in terms of heat transport.

### 7.1. Pressure reduction

A large number of experiments are reported in open literature on pressure reduction. Unfortunately many of the experiments have unrealistic boundary condition like for instance constant temperature. In many cases critical information are lacking in the reports. Some experiments are conducted without realistic sediments. Rather than attempting to discuss the varieties in experimental set-ups and associated experimental results it is better to focus on how the thermodynamic laws, as related to hydrate dissociation, are affected.

#### 7.1.1. Heat transport

In the first law that enters the energy balances a radial heat transport model can be written as:26
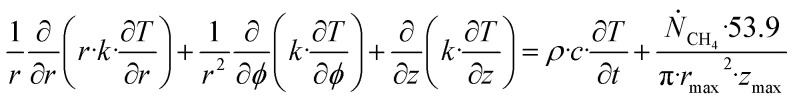


Accumulation of energy in the volume elements from a producing well is omitted. *r* is a radial distance from the producing well, *ϕ* is the a rotational angle and *z* is the height of the cylindrical transport section, *i.e.*: the height of the specific hydrate filled sediment section in consideration. *ρ* is density and *c* is specific heat capacity of the sediments containing hydrate, liquid water and gas. Approximating that the heat transport is uniform all around the cylindrical heat transport then there is no rotational dependency to the temperature and the second term vanishes. In a steady state limit then also the first term on the right hand side disappears. The temperature gradient in the last term on left hand side is the geothermal gradient and assumed to be approximately constant for the hydrate filled section. With all these simplifications then eqn (26) reduces to:27
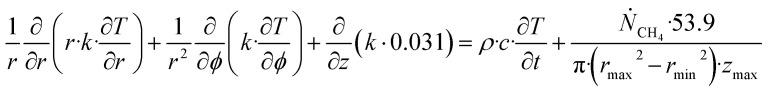


**Fig. 16:**
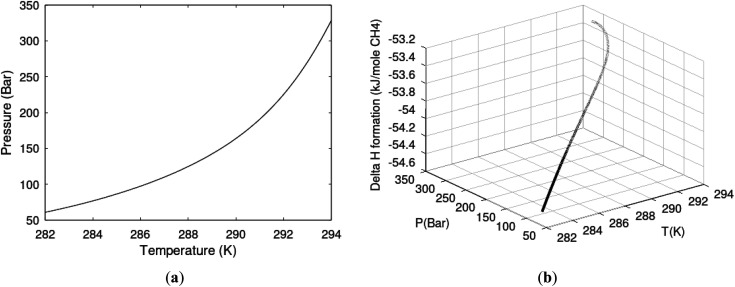
(a) Pressure temperature CH_4_ hydrate stability limits in Danobe paleodelta (b) Enthalpies of hydrate formation for CH_4_ hydrate along the pressure temperature hydrate stability in Danube paleodelta.

**Fig. 17:**
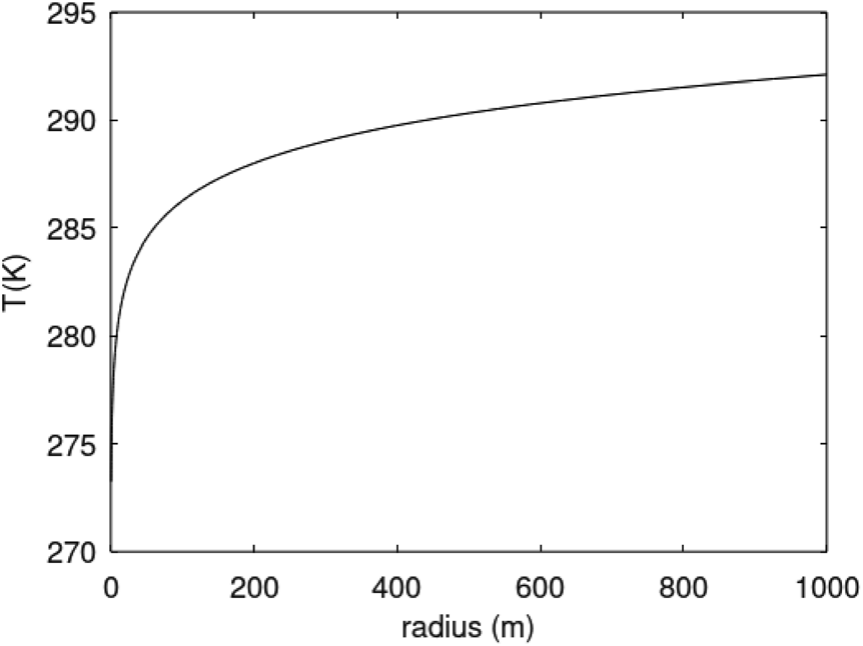
Temperature profile based on eqn (29) for *r*_max_ = 1000 m.

*Θ* is the geothermal gradient. For the Danube paleodelta values are reported to vary between 27 and 35 °C per km.^55^ A constant value of 31 °C per km is used in eqn (27). The enthalpies of formation for CH_4_ hydrate, in the relevant range of pressure temperature conditions for Danube paleodelta, are plotted in Fig. 16. The corresponding enthalpies of hydrate dissociation are the negative of the formation numbers along the pressure temperature hydrate stability limit. Since the hydrate dissociation occurs at the hydrate stability limit in pressure temperature projection then the pressure variable in (27) is an implicit function of *T* and integration will be in only *T* as function of *r*. The variation in enthalpies of hydrate dissociation for the actual conditions is limited and a constant average value of 53.9 kJ mol^−1^ CH_4_ is sufficient for the purpose, and rigor, of eqn (27). With constant *k* over the height of the hydrate filled sediment then the third term on the left hand side disappear. *r*_max_ is the maximum distance from the borehole that contributes significantly to a heat flow towards the borehole. Assuming that the borehole radius is available for gas production over the whole height, as an approximations, then *r*_min_ is the pipeline diameter. *z*_max_ is the height of the hydrate filled sediment. For stationary flow the first term of right hand side disappears (Fig. 17).28
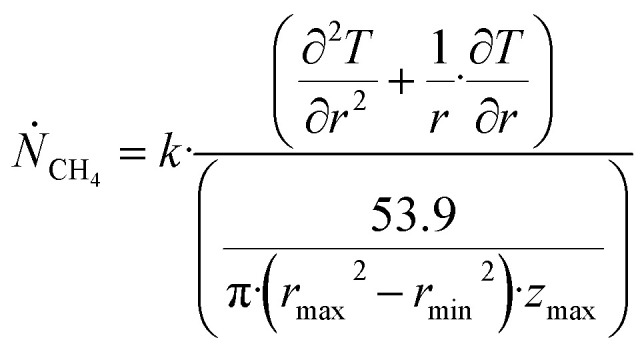


Recalculation to standard cubic meter is using ideal gas law. As a simple example consider the temperature profile in Fig. 17 as function of distance from a borehole with diameter 0.5 m and a radial extension of 1 km as a rather arbitrary number for efficient radial distance for heat supply to hydrate dissociation. Mathematically the plotted profile can be expressed as:

29 *T*(*r*) = e^[*a*_0_+*a*_1_ ln(*r*)]^

30
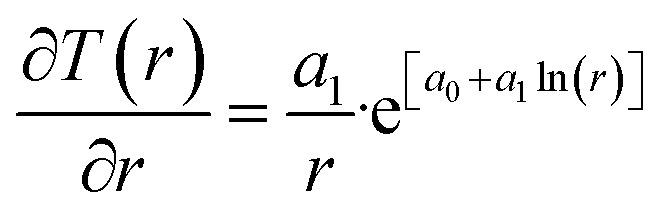
31
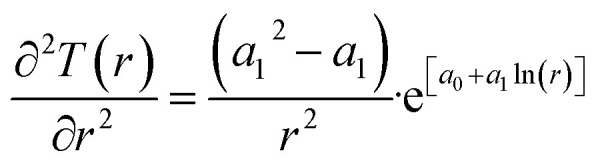


**Fig. 18:**
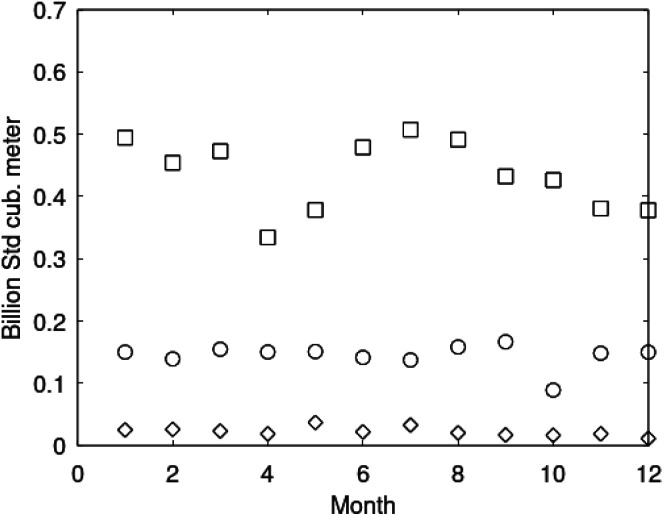
Monthly gas production rates from Kvitebjørn for 2008 (squares), Mikkel (circles) for 2005 and Lille-Frigg (diamonds) for 1997.

Parameters are given in Table 5. The selection is rather random and range from typical gas fields to oil mixed fields. Details on these data, and production data for fields offshore Norway, are openly available from Norwegian Petroleum Directorate.^66^ Monthly production rates for three fields are plotted in Fig. 18. See also Table 6.

**Table 5:** Heat transport limited production rates 
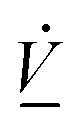
 in bcm per month for different radial extensions of significant heat transport towards producing well. Parameters *a*_0_ and *a*_1_ are the parameters in the temperature profile in eqn (29) for various maximum radius of significant heat transport interaction

*r*_max_ (m)	100	500	1000	5000	10 000
*a*_0_	5.6192	5.6171	5.6165	5.6154	5.6165
*a*_1_	1.2586 × 10^−2^	9.6538 × 10^−3^	8.7735 × 10^−3^	7.2404 × 10^−3^	6.7336 × 10^−3^
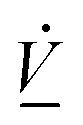	1.3 × 10^−5^	2.0 × 10^−4^	6.5 × 10^−4^	1.1 × 10^−2^	3.8 × 10^−2^

**Table 6:** Except for Sleipner, which is a small CO_2_ CCS case for removing CO_2_ from a hydrocarbon stream the three example cases from offshore Norway are based on the use of a CO_2_/N_2_ mixture containing 70 mol% CO_2_. The numbers for these three fields are based on 1.43 moles injection gas per mole CO_2_ utilized for CH_4_ production. The cost is based on a price of 0.01 US$ per kW h and the cost numbers are in thousands US$. kw is kilowatt, kW h is kilowatt hours. Mt y^−1^ is million tons per year. Temperature is 293 K and as simplified example temperature is constant. *W*_s_ is “shaft work”, pressures are in bar

*P*	*W*_s_	Sleipner			Lille-Frigg			Mikkel			Kvitebjørn		
		Mt y^−1^	mol s^−1^	kg s^−1^	Mt year^−1^	mol s^−1^	kg s^−1^	Mt year^−1^	mol s^−1^	kg s^−1^	Mt year^−1^	mol s^−1^	kg s^−1^
		1.274	1020	40	0.614	495	19	3.932	3180	124	11.836	9572	375
		kW	Bwh y^−1^	Cost per y	Kw	Bwh y^−1^	Cost per y	kW	Bwh y^−1^	Cost per y	kW	Bwh y^−1^	Cost per y
200	1.18	1204	10.55	105.5	584	5.116	51.16	3752	32.87	328.7	11 295	98.94	989.4
230	1.24	1265	11.08	110.8	614	5.379	53.79	3943	34.54	345.4	11 869	104.0	1040
250	1.26	1285	11.26	112.6	624	5.466	54.66	4007	35.10	351.0	12 061	105.7	1057
270	1.27	1295	11.34	113.4	629	5.510	55.10	4039	35.38	353.8	12 156	106.5	1065
300	1.28	1306	11.44	114.4	634	5.554	55.54	4070	35.65	356.5	12 252	107.3	1073

The profile in Fig. 17 is fairly random. Initial situations for real hydrate deposits vary from high hydrate saturations and no free gas, to low hydrate saturations and substantial free gas phase in pores. Pumping out water does not result is significant Joule–Thomson effects and associated radial temperature gradients may be very small. Exporting energy from a specific volume section of the reservoir will, however, lead to cooling of the heat supply source. It is beyond the scope of this work to make iterative calculation of extracted energy for the hydrate dissociation in Table 4 and subsequent adjustment of temperature profiles adjusted for exported energy.

The pressure reduction may generate temperatures below freezing. It is, however, unrealistic to produce gas with associated water for sub-zero temperatures and that is the reason for a minimum temperature of 273.15 K in Fig. 17.

292 K and lower temperatures are not sufficient to break hydrogen bonds efficiently. There is no available information on the dynamics of destabilization of hydrogen bonds at low temperatures in open literature. It is therefore not possible to evaluate second law requirements. There is some data on dissociation of ice exposed to air at various temperatures. Due to the differences in hydrogen bonded structures in ice and also the fact that hydrate also contains guest molecules that related to surrounding components thermodynamically.

Similarly there is also a lack of available information to evaluate impact of these low temperatures on the combined law.

The second mechanism that can lead to hydrate dissociation caused by pressure reduction is the chemical work. This is the last term on right hand side in eqn (2) for the first law and eqn (9) for the combined law. See Appendix B in ref. 3 for an example. Without specific data on mass fluxes through the hydrate filled sediments in a specific reservoir there is no basis for evaluation of this specific mechanism. Dissociation in guest chemical potential gradients is slow. Then again, however, as discussed above the efficiency of dissociating hydrate in low temperature gradients are also highly uncertain. The observed oscillating behaviour for the second test offshore Japan^16,17^ also illustrates the complexity of extracting low temperature heat for hydrate dissociation, and the additional effect of cooling down the heat source due to extracted hydrate dissociation energy.

### 7.2. Thermal stimulation

Adding heat is technically efficient. There are, however, a substantial number of ways to accomplish this and they are all very different as the way the heat is distributed. Adding steam exchanges steam condensation energy for hydrate dissociation and heat conduction throughout the reservoir. Hot water, potentially hot seawater, is a less costly option which also benefits from direct injection into pore space rather than a complex transition stage of condensing steam before liquid water enters pore space. Common to all these options is that they are very expensive and substantial amounts of heat are lost to heating minerals. STATOIL (now EQUINOR) concluded^68^ 10 years ago that thermal stimulation is not commercially feasible. No arguments are made whether this also holds today or not.

Every hydrate reservoir is unique in very many aspects. This includes the more or less stationary state due dynamics of hydrate dissociation from incoming water and creation of new hydrate from upcoming gas. Frequently this balance between dynamics of hydrate dissociation from top of hydrate filled sections, and creation of new hydrate from bottom of hydrate filled sediments is the main factor that determines hydrate saturations in the pores. Hydrate saturation and related initial flow dynamics in the reservoir is one of the factors that will determine efficiency of fluid injection and the efficiency of spreading fluids around in the hydrate filled sediments. Water is of course easier to inject than CO_2_/N_2_ mixtures discussed in the next section.

Given that the economic feasibility of heat addition is very uncertain for any characteristics of hydrate deposits it does not makes sense to go into details and/or review of methods for thermal stimulation in the context of this paper. Furthermore, the thermal stimulation method, if applied alone, reduces the stability of the working volume of the sediments, increasing the water content, since the dissociation of 1 volume of hydrate creates ∼0.8 volumes of water.

### 7.3. Chemicals

Adding electrolyte solutions containing different ions is clearly efficient in breaking hydrogen bonds. Corrosion issue and cost depends on types of salts used. Sodium chloride is inexpensive and likely economically feasible even though portions of injected saline water will be lost. The density of water is proportional to salt content and saline water will sink. It is also environmentally friendly. Technically the efficiency is proportional to ability to distribute the injection water through the hydrate filled sediments. The technology is simple and does not need any extensive discussion here.

**Fig. 19:**
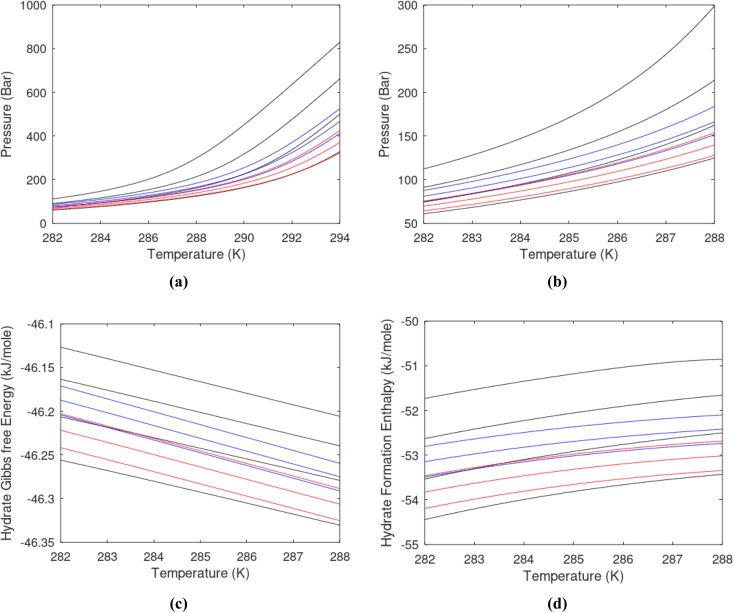
(a) Pressure temperature hydrate stability limits as function of mole-fraction inhibitor in water for CH_4_ hydrate. Lower black solid curve is for pure water. Black curves are (upward) for mole-fractions 0.02, 0.03 and 0.04 NaCl in water. Blue curves are (upwards) for mole-fractions 0.02, 0.03 and 0.04 methanol in water. Red curves are (upwards) for mole-fractions 0.02, 0.03 and 0.04 ethanol in water. (b) Same as (a) for a more narrow temperature region. (c) Hydrate Gibbs free energy for CH_4_ hydrate as function of mole-fraction inhibitor in water. Lower black solid curve is for pure water. Black curves are (upward) for mole-fractions 0.02, 0.03 and 0.04 NaCl in water. Blue curves are (upwards) for mole-fractions 0.02, 0.03 and 0.04 methanol in water. Red curves are (upwards) for mole-fractions 0.02, 0.03 and 0.04 ethanol in water. (d) Enthalpy of hydrate formation for CH_4_ hydrate as function of mole-fraction inhibitor in water. Lower black solid curve is for pure water. Black curves are (upward) for mole-fractions 0.02, 0.03 and 0.04 NaCl in water. Blue curves are (upwards) for mole-fractions 0.02, 0.03 and 0.04 Methanol in water. Red curves are (upwards) for mole-fractions 0.02, 0.03 and 0.04 ethanol in water.

Methanol and ethanol have excellent properties with water. They are solvable and move with water at comparable liquid water diffusivity coefficients. This is important in the behaviour towards hydrate/liquid water interface hydrogen bonds, as well as on hydrate surfaces. Methanol is, however, highly poisonous and will likely be banned for use in many relevant areas since it will pollute groundwater. Ethanol is more expensive but less poisonous. To our knowledge Brazil is the only country that utilizes ethanol for hydrate prevention since they produce low cost ethanol from sugar industry waste products. The thermodynamic inhibition aspects are well known over many decades of hydrate prevention research, and associated experimental work. Some few examples are, however, also included here since the thermodynamic model utilized in this work can also provide values for hydrate stability (Gibbs free energy) and hydrates dissociation enthalpy for use in first law energy balances. The thermodynamic models have been verified elsewhere^45,69^ for NaCl, as well as for alcohols, and not repeated. Fig. 19 is, however, unique since the conditions are different than the typical conditions that have been examined in earlier publications. And of course these also highly relevant for production of hydrates from Black Sea using CO_2_/N_2_ mixtures with alcohol as possible additional components for keeping the interface between injection gas and pore water free of blocking hydrate films. See also Section 7.4.

As expected the CH_4_ hydrates formed from water containing salt or alcohols are less stable (higher Gibbs free energy) than hydrates formed from pure water. CH_4_ hydrate formation enthalpies for hydrates formed are lower for two reasons. One reason is the effects of inhibitor on water but also the fact that higher pressures are needed to form hydrates from water containing inhibitors. The density of the guest molecules in the gas phase outside of the hydrate is therefore higher for the systems with inhibitor. Inclusion enthalpy difference is therefore lower for hydrates formed from water containing hydrate.

The examples in Fig. 19 are well known classical inhibitors that are solvable in water. Surfactants with surface exposed polar groups or even ionic surfactants are also alternatives that should be investigated further. High molecular weight surfactants, however, may not be the way to go since there will always be a risk of agglomeration and clogging. Nature is full of surfactants and there is much room for more research.

Hydrates in clay and very fine sand are typical systems that might be considered for chemical hydrate production. Black Sea hydrates are conventional hydrates in coarse grain sand which does not need complex and expensive methods to release natural gas from hydrates.

### 7.4. CO_2_ injection

There are very many experimental papers on the use of CO_2_ for combined CO_2_ storage and release of CH_4_. Every research group have their own design of experimental set-up. Frequently critical data are missing in the published report from experiments. Often the boundary conditions are inappropriate since they interfere with the mechanism for the CO_2_/CH_4_ swapping that is the purpose of the exchange. One example is experiments that are conducted at constant temperature. Since heat released from formation of a new hydrate from injection gas is a primary mechanism for the CO_2_/CH_4_ swapping it is obvious that temperature control of the experiment does not make sense. Instead of discussing a substantial number of different experimental set-ups and many different boundary conditions we skip any review of experimental efforts, including our own experimental efforts during latest 3 decades. The main scientific method in this work is classical thermodynamics analysis, based on the fundamental laws of thermodynamics. Within that perspective there are few or actually none other relevant papers to discuss and refer to except our own. There are indeed very many good papers on hydrate published every year. We are, however, the only group that utilize a totally uniform thermodynamic reference system for hydrate formed and dissociated through any possible route. Through several papers we have discussed heterogeneous hydrate formation on gas/liquid water interface as well as homogeneous hydrate formation from guest molecules dissolved in water. Lately we focus more on hydrate nucleation towards mineral surfaces. The advantage of residual thermodynamics is that the reference state is the same as ideal gas space during samplings from Molecular Dynamics (MD) simulations. With appropriate models for chemical potentials of water and guest in adsorbed state there are modern algorithms for efficient sampling of chemical potentials. See for instance Kvamme *et al.*^1^ for example of CH_4_ hydrate nucleation towards mineral surface. Ref. 70–75 provides overview of modern adsorption studies in classical MD modelling, including calculations of chemical potentials for adsorbed water and adsorbed guests, as well as for adsorbed ions and polar components. This is also relevant for injection of CO_2_/N_2_ with addition of alcohol and/or surfactant.

Injection of CO_2_ into hydrate filled sediments requires two different types of additives. The first additive is a component that can increase injection gas permeability. The ideal choice is a component that can enter hydrate while being inferior to CO_2_ in large cavity occupancy. A typical choice can be N_2_ or even air. The second additive is an additive that can prevent hydrate blocking films on the interface between injection gas, and pore water. Low molecular surfactants are natural choices.^7,76,77^ Small alcohols, like methanol and ethanol, have surfactant properties^6,45,69,78^ due to low polarity parts related to methyl groups. The advantage of these small alcohols is that they move efficiently together with water on interfaces. In contrast to the first type of additives these are added in very small amounts based on an interface area action mechanism.

The total commercial income to the concept consists of the CO_2_ emission reduction value. Different countries have various ways to set a value on this. Norway, as an oil and gas producing country, started with a tax of 51 US$ per ton CO_2_ emission from oil and gas production back in 1991. This has later changed to a tax per produced unit of gas and oil respectively. The initial tax was the economic motivation for the CO_2_ storage in Utsira back in 1996. STATOIL (EQUINOR) philosophy was that one million ton CO_2_ per year separated from the Sleipner hydrocarbon system should be stored in underground aquifers at a cost lower than the CO_2_ emission tax. This project is still running and the same motivation was behind CO_2_ storage from Snøhvit (0.7 million ton CO_2_ per year). Looking at some large energy consumers like Germany the CO_2_ tax is 25 Euro per ton CO_2_ from 2021 and supposed to increase to 55 Euro per ton CO_2_ in 2025.^76^

Technically there are only minor differences in using air rather than N_2_. Oxygen will have slightly higher attraction to water than N_2_ and as such no negative impact. If any cost of adding air is disregarded then the “sales value” of accepting CO_2_ from customers will be set to 55 Euro per ton. At this stage it is unknown what Yara in Netherlands is paying Northern Lights for storing 0.8 million ton CO_2_ per year from ammonia production^76,77^ in underground aquifers offshore Norway.

Released CH_4_ after the swapping will migrate upwards due to buoyancy and can be directed towards a network of receiving wells. New drilling technology (ref. 2 and references therein) reduces the cost of well drilling substantially as compared to conventional drilling technology. This makes it feasible to optimize a network of receiving wells kept at pressures slightly lower than local static pressure but still high enough to reduce transport of liquid water with the sampled gas to a minimum.

**Fig. 20:**
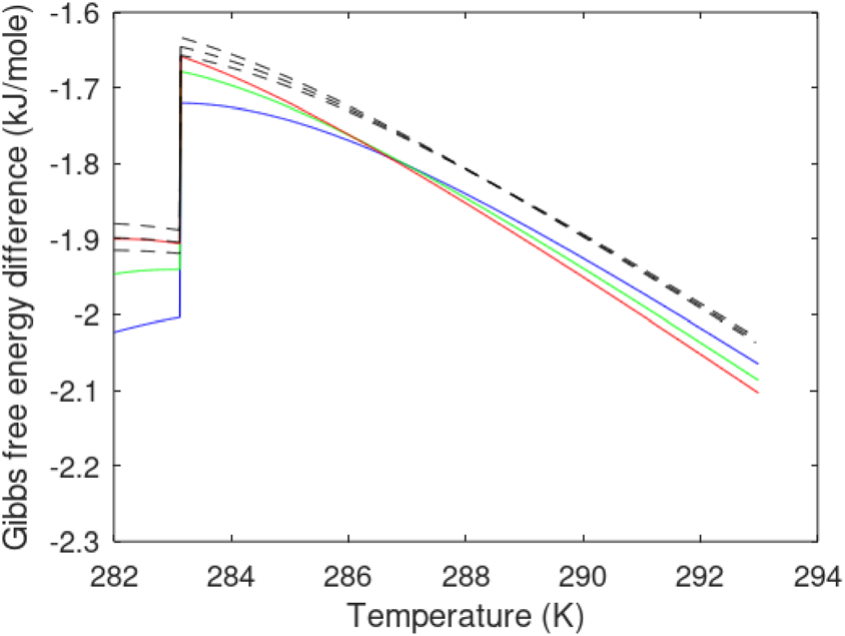
Gibbs free energy difference between CO_2_ mixtures and pure CH_4_ hydrate. Hydrate from 10 mol% N_2_ in CO_2_/N_2_ mixture (green solid), hydrate from 20 mol% N_2_ in CO_2_/N_2_ mixture (red solid), hydrate from 30 mol% N_2_ in CO_2_/N_2_ mixture (blue solid). Upper dashed black curve is for a mixture of 69 mol% CO_2_, 30 mol% N_2_ and 1 mol% CH_4_. Next dashed black curve is for a mixture of 75 mol% CO_2_, 24 mol% N_2_ and 1 mol% CH_4_. Lowest dashed black curve is for a mixture of 80 mol% CO_2_, 19 mol% N_2_ and 1 mol% CH_4_.

The cost of compressing the gas mixture is trivially the enthalpy difference between the inlet condition and the outlet condition as corrected for entropy losses. In usual compressor design the entropy losses are incorporated through an efficiency percentage. Practically the entropy losses increase the finale temperature but then the fluid is transported to the injection point and expected to exchange heat with surroundings. Just as a conservative example we use an inlet condition of atmospheric pressure and temperature 293 K. Without the usual iteration on zero entropy change and then subsequent correction for efficiency an outlet fluid temperature of 298 K may serve as good enough as example. Cost of gas compression for some injection rates ranging from storage of small point sources like for instance Sleipner (1 million ton CO_2_ per year), which is also representative for the point source from Yara mentioned above as well as the initial point source in Northern Lights. That is a point source of 1.3 million ton CO_2_ per year from cement industry in south eastern Norway. 1 mole CO_2_ injected will theoretically release more than 1 mole of CH_4_.^48^ Nevertheless – as rough conservative estimate in Table 6 below we use that 1 mole CO_2_ injected releases 1 mole CH_4_. Production rates in Fig. 20 is for an ideal gas reference state and mole numbers of CO_2_ needed to produce those levels of CH_4_ production is trivially proportional. Maximum N_2_ mole-fraction in injection gas is set to 0.3 and moles injection gas per mole CO_2_ injected is therefore 1.43 moles. The numbers in Table 6 for the example fields in Fig. 20 are amounts of CO_2_/N_2_ injection gas needed to release those rates of CH_4_. Calculation of shaft work is generally an iterative process in which the entropy change (and associated temperature change) is an iterative solution based on a given efficiency in conversion of the necessary work needed for the compression. This is fairly trivial in chemical engineering calculations and does not need detailed explanations here. For orders of magnitude calculation in the context of this section isothermal estimates are sufficient for illustration. It is not officially known what Yara Netherlands are going to pay the Northern Lights project for storing 0.8 million tons CO_2_ per year in underground aquifers offshore Norway. If we use 50 US$ per ton CO_2_ based on level of CO_2_ taxes in various countries in Europe it is obvious that the compression costs are very low compared to the net income of receiving the CO_2_ for storage. The cost of nitrogen is low and can be replaced by the use of air, which then can be considered as zero cost. A very minor cost of keeping CH_4_ receiving wells at slightly lower pressures than local static pressure is not significant. Only difference in investment costs as compared to aquifer storage is these receiving wells. New drilling technology (see reference in Kvamme & Saeidi^2^) reduces this cost substantially as compared to conventional drilling technology.

For CO_2_/N_2_ to be feasible there are 4 criteria that has to be met:^48^

(1) Temperature pressure hydrate stability limits for hydrate from injection gas (CO_2_ with N_2_ and possible additives) has to be below the corresponding curve for *in situ* CH_4_ hydrate.

(2) Gibbs free energy of the hydrate formed from injection gas has to be lower (more negative) than Gibbs free energy for *in situ* CH_4_ hydrate.

(3) The enthalpy of hydrate formation for injection gas hydrate has to be higher in absolute value than the absolute value of hydrate formation enthalpy for *in situ* CH_4_ hydrate.

(4) The level of temperature supplied to *in situ* hydrate is high enough to break hydrogen bonds and provide the necessary entropy change from low entropy in solid hydrate to higher entropies for corresponding amounts of liquid water and gas.

**Fig. 21:**
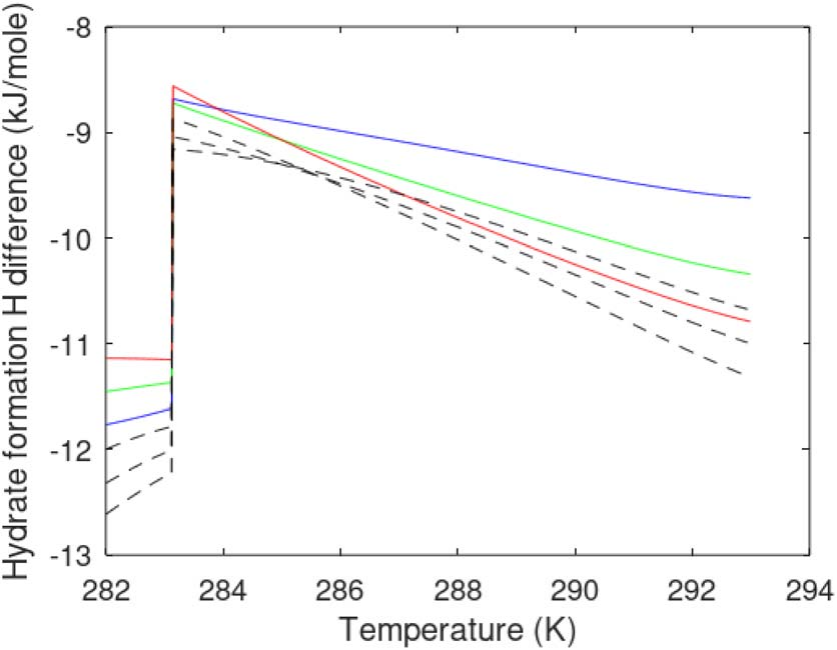
Enthalpy of hydrate formation between CO_2_ mixtures and pure CH_4_ hydrate. Hydrate from 10 mol% N_2_ in CO_2_/N_2_ mixture (green solid), hydrate from 20 mol% N_2_ in CO_2_/N_2_ mixture (red solid), hydrate from 30 mol% N_2_ in CO_2_/N_2_ mixture (blue solid). Dashed black curve is for a mixture of 69 mol% CO_2_, 30 mol% N_2_ and 1 mol% CH_4_. Upper dashed black curve is for a mixture of 69 mol% CO_2_, 30 mol% N_2_ and 1 mol% CH_4_. Next dashed black curve is for a mixture of 75 mol% CO_2_, 24 mol% N_2_ and 1 mol% CH_4_. Lowest dashed black curve is for a mixture of 80 mol% CO_2_, 19 mol% N_2_ and 1 mol% CH_4_.

Criteria (2) is always met, as discussed elsewhere,^1–3,26,29,44,45,78^ for CO_2_ and mixtures of CO_2_ with N_2_ up to roughly 25 mol% N_2_. 30 mol% N_2_ is feasible because some CO_2_ will distribute to other phases (dissolved, adsorbed on minerals, adsorbed on water, adsorbed on hydrate) and some N_2_ may even degas from CO_2_/N_2_ mixture during dynamic phase transitions. Experiments^79^ indicate practically no difference in performance between injection of CO_2_/N_2_ mixture containing 20 mol% N_2_ and 30 mol% N_2_. Illustrations of Gibbs free energy differences between injection gas and *in situ* CH_4_ hydrate are plotted in Fig. 21. All the mixtures are relevant. The addition of small amount of CH_4_ is very favorable for stabilization of the small cavities, even though also N_2_ will provide some hydrate stabilizing effect by filling the small cavities.

Criteria (3) is met for N_2_ additions up to 30 mol% in CO_2_/N_2_ injection mixture.^1–3,26,27,29,44,45,78^ See also Fig. 21 below, which is for typical Black Sea conditions.

Hydrate formation for all the mixtures can serve as efficient heat source for dissociation of *in situ* CH_4_ hydrate.

The primary mechanism for CO_2_/CH_4_ swapping in liquid water region of temperatures is the utilization of hydrate formation enthalpy from formation of injection gas hydrate and transfer of heat, on a pore scale, to *in situ* CH_4_ hydrate. Fast heat transfer through water phases in pores^80^ is likely to provide high enough temperatures when the released heat from injection gas hydrate formation reaches *in situ* CH_4_ hydrate in the pores. This is particularly true since *in situ* hydrate is filling the inner pore volume and injection gas enters space between hydrate and sediment walls of the pores. It is therefore expected that criteria (4) is met. It is fully possible to evaluate this assumption using heat conduction models in eqn (7) and integration between appropriate boundary conditions for different hydrate saturations (and corresponding free liquid water distribution in the pores).

**Fig. 22:**
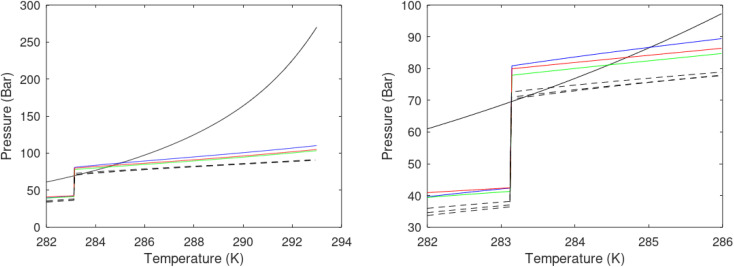
Pressure temperature hydrate stability limits for pure CH_4_ hydrate (black solid), hydrate from 10 mol% N_2_ in CO_2_/N_2_ mixture (green solid), hydrate from 20 mol% N_2_ in CO_2_/N_2_ mixture (red solid), hydrate from 30 mol% N_2_ in CO_2_/N_2_ mixture (blue solid). Upper dashed black curve is for a mixture of 69 mol% CO_2_, 30 mol% N_2_ and 1 mol% CH_4_. Next dashed black curve is for a mixture of 75 mol% CO_2_, 24 mol% N_2_ and 1 mol% CH_4_. Lowest dashed black curve is for a mixture of 80 mol% CO_2_, 19 mol% N_2_ and 1 mol% CH_4_.

Criteria (1) is a bit more special since the hydrate formed from injection gas may not be in contact with *in situ* CH_4_ hydrate. If reformation of hydrate becomes an issue then criteria (1) might be an issue but not necessarily since the density of released CH_4_ is relative low and will lead to CH_4_ migration upwards by buoyancy and towards collecting wells by pressure gradients. From a mass availability in the pores it is more likely that CO_2_ is present in separate phase. Fig. 22 is a plot of the pressure temperature hydrate stability projection for the conditions also applied in Fig. 20 and 21.

**Fig. 23:**
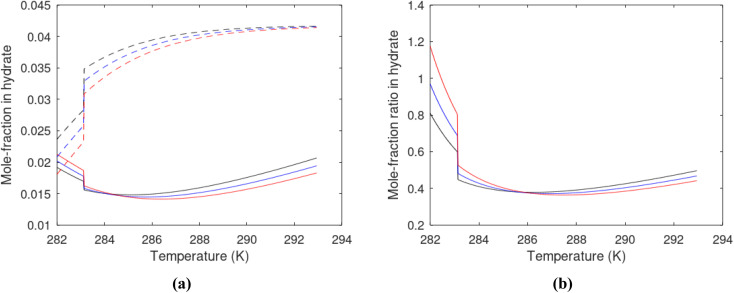
(a) Mole-fractions CH_4_ in hydrate (solid) and N_2_ in hydrate (dashed). Black curves are for a mixture of 69 mol% CO_2_, 30 mol% N_2_ and 1 mol% CH_4_. Blue curves are for a mixture of 75 mol% CO_2_, 24 mol% N_2_ and 1 mol% CH_4_. Red curves are for mixture of 80 mol% CO_2_, 19 mol% N_2_ and 1 mol% CH_4_. (b) Ratios between mole-fractions CH_4_ and N_2_ in hydrate. Black curves are for a mixture of 69 mol% CO_2_, 30 mol% N_2_ and 1 mol% CH_4_. Blue curves are for a mixture of 75 mol% CO_2_, 24 mol% N_2_ and 1 mol% CH_4_. Red curves are for mixture of 80 mol% CO_2_, 19 mol% N_2_ and 1 mol% CH_4_.

Just to illustrate the favorable stabilization of the small cavities for even only and addition of 1 mol% CH_4_ the hydrate mole-fractions of CH_4_ and N_2_ are plotted in Fig. 23. Despite a gas mole-fraction ratio of CH_4_ to N_2_ in gas ranging from 1/30 to 1/19 the ratio between the two components in the hydrate reflects the superior stabilization of the small cavity by CH_4_ as compared to N_2_, which is as expected.

Nevertheless – when choosing gas for mixing with CO_2_ then N_2_ or air is a reasonable choice since N_2_ and O_2_ at least provide some stabilization of small cavities. This is in contrast to H_2_. The reference to experimental studies on the use of H_2_ as permeability increasing additive is not given here since the study lacked from many important details in the publication. Another interesting aspect of N_2_ and O_2_ is the possibility of injecting flue gas directly. Natural gas power plants typically utilize fairly large amounts of excess air in order to ensure efficient combustion of the gas. The mol% N_2_ and O_2_ in the exhaust mixture may therefore exceed the “magic” 30 mol% discussed here. The differences in water solubility between CO_2_ and these two gases make it feasible to design coarse grained separation at low pressures without the use of costly amine solutions. Specific proposals on this are available but beyond the scope of this work. Other combustion situations may be more favorable for direct compression and injection.

The addition of 1 mol% CH_4_ is favorable for all the injection gas properties that are needed. This addition is, however, most important for the changes in pressure temperature hydrate stability limit regions. If the local relevant range of conditions (with reference to Fig. 22), or other aspects does not make it necessary to add CH_4_ then this cost can certainly be avoided.

In summary there is nothing very special about injection of CO_2_/N_2_ for CO_2_/CH_4_ swapping as compared to injection for aquifer storage. Injection gas permeability is different because available liquid water in the pores is smaller. Or put in a different way – if porosity is defined by fluid volume per sediment volume unit, then porosity is low for hydrate filled sediments. Minerals are water wetting and in aquifer storage the injection of CO_2_ faces permeability challenges if CO_2_ density is high, like in the Snøhvit project.^81–84^ For the injection into hydrate filled sediments the primary role of the N_2_ is to dilute the CO_2_ and reduce the mixture density, and as a consequence also increase injection gas permeability. To a limited extent the presence of N_2_ will also disturb the establishment of hydrate blocking films. Efficient reduction of flow blocking hydrate films caused by injection gas hydrate films needs surfactant additives. Small alcohols like methanol and ethanol have surfactant properties in interface between aqueous phase and a nonpolar phase due to the methyl groups. In contrast to conventional surfactants these small alcohols are dissolvable in water. In the interface between gas and liquid water there will be two limiting situations. Closer to the gas the concentration of alcohol is large relative to water and the alcohol is the solvent for water. This will efficiently prevent hydrate formation in that part of the interface. Further into the liquid water side then water is the solvent for alcohol and hydrate can form at locations in which the activity of water permits. The transition of solvent between water and alcohol respectively is visible in different mixture properties. One of them is the dielectric constant. For water methanol mixtures this transition is illustrated by Fig. 13 in Kvamme^85^ in which the change in gradient for the dielectric constant reflect the change in which molecule that is the solvent and which is the solute.

The change in interface free energy due to the presence of alcohol increases the transport rate of hydrate formers into the liquid water side, as well as increasing the supersaturation of hydrate formers in the water interface. The presence of surfactant also increases the extension (width) of the interface.^6^

Large conventional surfactants may result in surfactant clogging that can also partly block the pores. Small molecules with surfactant effects may, however, be efficient. Morpholines is one class of possible surfactant^7,76,77^ in addition to small amounts of alcohol. On a molecular scale small alcohols moves (molecular diffusion) together with water in the interface with comparable diffusivity coefficients. Morpholines, and other small molecules with surfactant properties, are more stationary in the interface region and may have a stronger effect in keeping the interface open and free from blocking hydrates. The balance between alcohol and other types of surfactant active molecules, as well as the total amount of interface active additives, is one of the challenges that need further experiments in order to optimize CO_2_/CH_4_ swapping schemes.

Steam cracking has developed quite much since the early invention by Norsk Hydro back in 1913. The main developments have been on new and improved catalysts, and improvement in thermodynamic efficiency through variations in excess steam. The total conversion is a two stage reaction from CH_4_ to CO and then CO to CO_2_. CO_2_ is a large molecule that normally only fits into the large cavity of structure I. There are some exceptions for hydrates formed at temperatures far below zero and atmospheric conditions. Another example are CO_2_ hydrae formation from aqueous solutions containing chemicals that result in very high CO_2_ solubility and homogeneous CO_2_ hydrate formation. With only filling of large cavities them the CO_2_ mole-fraction in hydrate is in the order of 0.11. In contrast the mole-fraction CH_4_ in *situ* CH_4_ hydrate is in the order of 0.14. The ratio of released CH_4_ per mole new hydrate formed from injected CO_2_ is therefore higher than one. This opens up for a self-driven energy cycle in which the CO_2_ from cracking of produced CH_4_ is re-injected into the reservoir for release of more CH_4_ for production of H_2_ as the only net export product. This opens up for development of new areas in permafrost regions, in addition to clean development of offshore hydrate resources.

Hydrates is the Black Sea was analyzed by Kvamme & Vasilev^85,86^ in terms of possible injection gas mixtures for combined CO_2_ storage and CH_4_ production. Offshore hydrates around the world dissociate due to inflow of seawater through fracture systems. The reason is the CH_4_ concentration in the incoming seawater is normally close to zero and thus below the hydrate stability limit in Fig. 5. Kvamme and Vasilev^88^ analyzed some Black Sea systems as well as some systems offshore Norway (Nyegga) with a focus of whether injection of CO_2_ dominated mixtures could reduce leakage fluxes of CH_4_ to the oceans and potentially then also to air. In either case these fluxes increase the carbon content of the oceans.

**Fig. 24:**
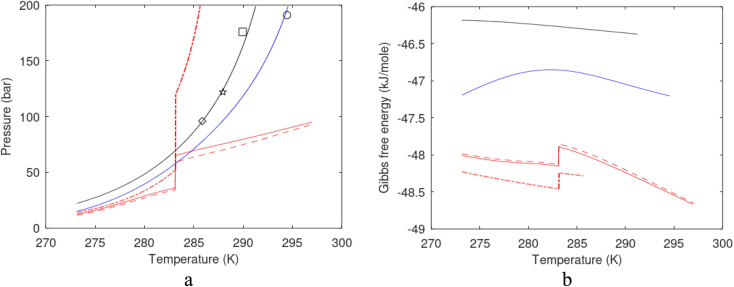
(a) Pressure temperature stability limits for some hydrates from Danube and Nyegga and model systems. Circle and square are from Danube while diamond and pentagram are from Nyegga. Solid black curve is calculated hydrate stability limits for pure CH_4_ hydrate while blue curve is calculated hydrate stability limits for a mixture of 1.75 mol% H_2_S and rest CH_4_. The two points from Nyegga and the highest (closest to seafloor) point from Danube appear to be close to pure CH_4_ hydrates while the deepest one from Danube might contain H2S based on the calculations as well as observations of H_2_S containing hydrates in the Black Sea.^85,86^ Red dash dot curve is for hydrates from pure CO_2_. Solid red is for a mixture consisting of 1 mol% CH_4_, 2 mol% C_2_H_6_, 70 mol% CO_2_ and 27 mol% N_2_. This injection mixture is denoted as mixture 1. Dashed red is for a mixture of 1 mol% CH_4_, 4 mol% C_2_H_6_, 70 mol% CO_2_ and 25 mol% N_2_. This injection mixture is denoted as mixture 2. (b) Gibbs free energy for the hydrates in (a).

The pressure temperature corresponding to depths for two hydrate sections at Danube^86–88^ and two hydrate sections from Nyegga^88^ are plotted in Fig. 24 below along with corresponding free energies and some possible injection mixtures.

Commercial use of the deepest Danube resource that likely contains significant amounts of H_2_S is not interesting. Although mixture 2 (Fig. 24) will be able to swap also that hydrate, and released H_2_S likely mix into new hydrate formation together with injection gas we focus on the three other systems. Highest temperature is therefore 289.95 K for the Danube system following the CH_4_ hydrate curve.

*In situ* CH_4_ hydrate is structure I hydrate and so is hydrate from injection gas. In terms of performance the relevant numbers are moles CH_4_ per mole water in hydrate, and mole CO_2_ in hydrate formed from injection gas per mole water.

For pure CH_4_ hydrate moles CH_4_ per mole water is simply:32
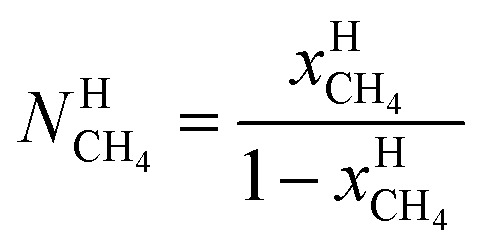


The mass balance for a mixture is also trivial and the linear algebraic equation for mole numbers of different guests in hydrate per mole water in hydrate is simply the solution to eqn (33) below.33
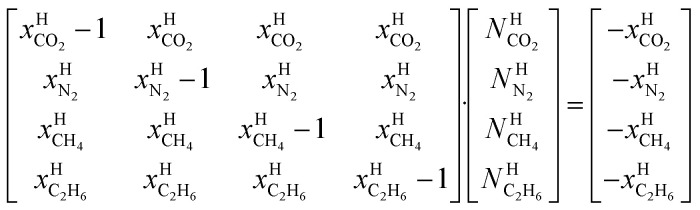


**Fig. 25:**
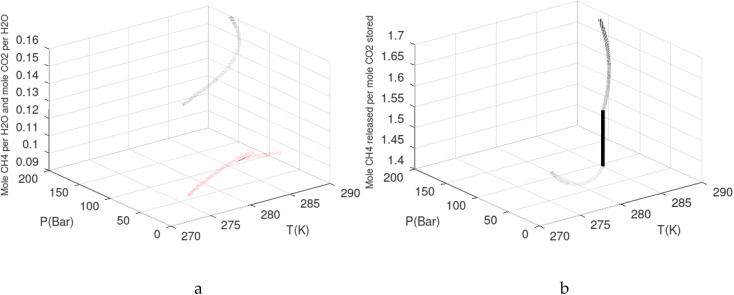
(a) Moles CH_4_ per mole water in *in situ* CH_4_ hydrate (black) and moles CO_2_ per mole water in hydrate formed from injection mixture 1 (red). (b) Moles CH_4_ released per mole CO_2_ stored as hydrates.

Moles CH_4_ in the *in situ* CH_4_ hydrate per mole water is plotted is plotted in Fig. 25 below along with moles CO_2_ in hydrate formed from mixture 1.

**Fig. 26:**
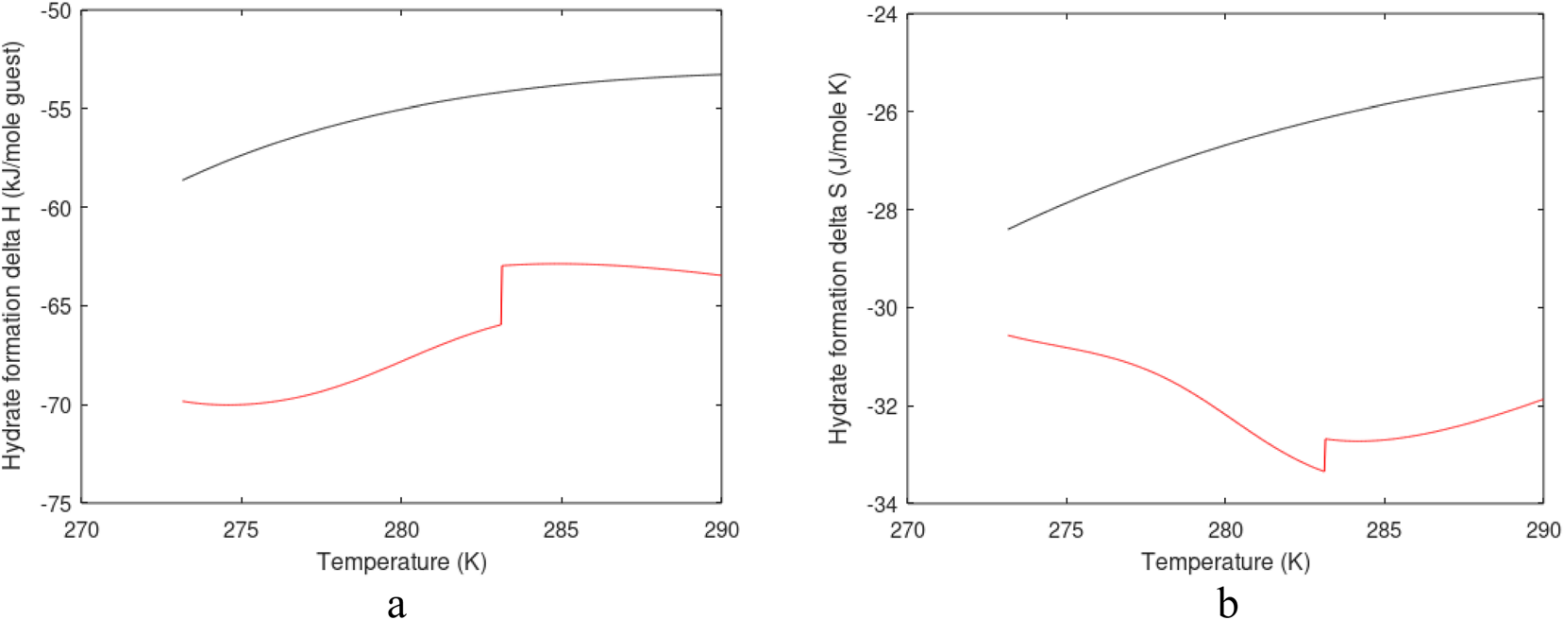
(a) Enthalpy of hydrate formation for hydrate from pure CH_4_ (black) and for hydrate from injection mixture 1 (red) in units of kJ mol^−1^ guest. (b) Entropy change of hydrate formation in J mol^−1^ K^−1^.

Just to complete the thermodynamic analysis for Danube and Nyegga CH_4_ hydrates the enthalpy change for hydrate formation for *in situ* CH_4_ hydrate, and for injection gas mixture 1, are plotted in Fig. 26 below along with the entropy changes for the hydrate formations. The enthalpy of hydrate formation for the injection gas is more than sufficient to dissociate the *in situ* hydrate even if it would have been a situation of 1 mole water creating hydrate from injection gas and needing to dissociate *in situ* CH_4_ hydrate to release 1 mole of water. For Danube hydrate systems, as well as for Nyegga hydrate systems, however the situation is far from these limitations in free pore water. Low hydrate saturation ensures availability of water for creation of significantly more than 1 mole injection gas hydrate per *in situ* CH_4_ hydrate that needs to be dissociated from the released heat of new hydrate formation.

Production of the released CH_4_ and direct use as energy source is an option but an even better option is to combine the swapping with steam cracking of released CH_4_. Ideally (100% conversion efficiency) each CH_4_ produce 2 moles of H_2_ for energy use and 1 mole of CO_2_ for re-injection back into the reservoir after makeup with the additional gas components in mixture 1. 100% conversion is of course not realistic but the actual conversion efficiency depends on the specific version of steam cracking. It is therefore better to leave the number as they are with 100% efficiency in the context of this work so that everyone can scale down based on the efficiency of the version used. Produced moles H_2_ per moles CO_2_ stored is then simply twice the numbers of released H_2_ in Fig. 25b. For the highest Danube section one mole CO_2_ stored as hydrate can ideally produce 3.36 moles H_2_. For Nyegga the two sections can produce 3.20 and 3.28 moles H_2_ per mole CO_2_ stored as hydrate respectively, for highest and deepest.

H_2_ as net export product is by itself a great value in developments towards a more sustainable future. In some regions of the world, however, the CH_4_/CO_2_ swap coupled with steam cracking into a cycle as described can provide new development of areas that may be fairly non-productive because they are remote from conventional energy sources. One of numerous examples can be found in Chinese permafrost regions.^90^

Finally it should also be mentioned that the steam cracking method is not limited to CH_4_. There are several versions that documents cracking of larger hydrocarbons. Some of the worldwide hydrates are of thermogenic origin, or a mixture of biogenic and thermogenic hydrocarbons. Typically we are then talking about structure I and structure II hydrates with up to iso-butane. Structure H can contain larger hydrocarbons but are very rare to find in nature.

## 8. Some estimates of commercial value of Black Sea hydrates

As discussed in Section 7.1 there are substantial challenges in extracting heat from surroundings over long time scales. And the fact that it is low temperature heat is a challenge in efficiently breaking hydrogen bonds and generating the necessary change in entropy from an ordered structure like hydrate over to disordered liquid water phase and even more disordered gas phase. Pressure change through heat supply from surrounding is generally questionable for the Black Sea hydrate saturations discussed in Section 5. If chemical work in the last term on right hand side of first law, and the combined law in terms of free energy, is to provide an efficient production mechanism the water inflow from surroundings have to come from sections of the sediments which does not contain hydrate. There is no information in the data reported in Section 5 on whether this is realistic or not.

Until it can be verified that the limitations in heat supply, and limitations in chemical work as production mechanism, can be overcomed then pressure reduction method may not provide any long term natural gas value from Black Sea hydrates. This statements is also supported by the failures of the two pressure reduction tests offshore Japan.^15,16^

Adding heat in the forms of steam, hot water or other methods of direct heat supply is not expected to be cost efficient as discussed in Section 7.2. Limited hydrate saturation involved in fairly large heat loss in heating water and minerals surrounding the hydrate. Low hydrate saturations is also a substantial challenge for the effect of adding water soluble chemicals due to the *in situ* dilution of chemicals in pore water. Exceptions are surfactants which are active on liquid water surface and can be efficient in turbulent flow situations when polar or ionic groups collides with hydrate surfaces. One challenge might be that some surfactants might adsorb on mineral surfaces and reduce amounts of surfactant available for action on hydrate surfaces. To our knowledge it has never been verified if this category of chemicals are dynamically efficient in producing hydrates.

The nano scale limitations of mass transport across the hydrate/liquid water interface is a bottleneck that also controls macroscopic hydrate phase transition dynamics. This bottleneck appears in hydrate growth as well as hydrate dissociation. As such eqn (24) in Section 4.2.2 applies to hydrate dissociation as well, with an opposite sign for hydrate dissociation relative to hydrate growth. The thermodynamic control factor in eqn (20) is of course different qualitatively as well as quantitatively. The qualitative difference is that hydrate nucleation and growth contains and “push-work” penalty for giving space for the new phase. This is typically not present in hydrate dissociation. Quantitatively Gibbs free energy change in the thermodynamic factor is sensitive to the actual temperature. The associated heat transport term is proportional to the temperature of the water surrounding the *in situ* CH_4_ hydrate as a result of the released heat due to formation of a new hydrate from the injection gas. Within all levels of uncertainties related to reservoir and all related characteristics, including hydrate saturation we might as well set the thermodynamic factor to unity without any justification beyond the level of uncertainties. Fixing the mole-fraction CH_4_ in the hydrate to 0.14 as an approximation we end up with a model for CH_4_ hydrate dissociation flux.34
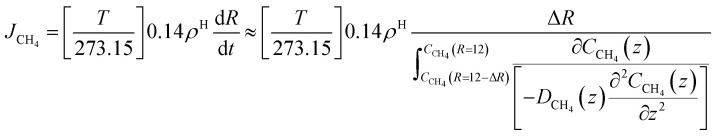


Eqn (26) is not new and was developed and utilized in Kvamme *et al.*^21^ to predict hydrate film thickness for CH_4_ hydrate and CO_2_ hydrate respectively. Agreement with experimental observations were remarkably good. As such the model has been verified elsewhere.^21^

At this level of available information on the reservoir, and uncertain in hydrate saturations and pore scale properties, it is only possible to provide some best possible estimates of CH_4_ release. This can be done for some values of temperature, and the assumption that hydrogen bonds in the liquid water/hydrate interface is efficiently broken and that the hydrate dissociation delay due to interface transport is zero. We also assume that the addition of N_2_ is sufficient to avoid bottlenecks relate to injection gas permeability. And furthermore we will assume that added surfactant or surfactant mixture (for instance alcohol and classical surfactant) is sufficient to avoid blocking hydrate films during formation of hydrate from injection gas.

The molecular transport during a dynamic hydrate dissociation may be significantly faster than self diffusion in liquid water. Estimated hydrate dissociation flux in moles CH_4_ m^−2^ s^−1^ as function of temperature and diffusivity coefficient are listed in Table 7 below.

**Table 7:** Flux (billion standard m^3^ CH_4_ m^−2^ per month) as function of temperature and liquid diffusivity coefficients in eqn (21)

*D*_liq_ *T*	1 × 10^−8^ m^2^ s^−1^	5 × 10^−8^ m^2^ s^−1^	1 × 10^−7^ m^2^ s^−1^	5 × 10^−7^ m^2^ s^−1^	1 × 10^−6^ m^2^ s^−1^	5 × 10^−6^ m^2^ s^−1^
	Flux	Flux	Flux	Flux	Flux	Flux
274	6.54 × 10^−5^	3.26 × 10^−4^	6.54 × 10^−4^	3.26 × 10^−3^	6.54 × 10^−3^	3.26 × 10^−2^
278	6.75 × 10^−5^	3.37 × 10^−4^	6.75 × 10^−4^	3.37 × 10^−3^	6.74 × 10^−3^	3.37 × 10^−2^
282	6.95 × 10^−5^	3.48 × 10^−4^	6.95 × 10^−4^	3.47 × 10^−3^	6.95 × 10^−3^	3.48 × 10^−2^
286	7.15 × 10^−5^	3.58 × 10^−4^	7.15 × 10^−4^	3.57 × 10^−3^	7.16 × 10^−3^	3.58 × 10^−2^

The range maximum temperature in Table 7 might be low and is limited by the models for diffusivity and concentration profiles. Extrapolarions to higher temperatures using the similar scaling factor in eqn (26) is expected to be fair though. It is rather unclear what the actual hydrate surface temperature is since the consumption of heat for breaking hydrogen bonds in water/hydrate interface and during dissociation of hydrate leads to cooling of the hydrate core. Some published Phase Field Theory (PFT) simulations^11,89^ provides insight into possible temperature distributions on CH_4_ hydrate core during CO_2_/CH_4_ swapping.

The conversion of the flux numbers from Table 7 to expected production rates goes through expected efficient area covered by injection gas. This is a technological issue on how the injection is performed as well as flow and distribution aspects in the balance between the properties of the injection gas, hydrate saturation of the *in situ* CH_4_ as well as many other properties that dictate flow and fluid distributions. It is also feasible to inject through several wells, which may also be economically feasible with new low-cost drilling technology. See Kvamme and Saeidi^2^ for references and discussion on that. Within the level of information that is available on the Black Sea hydrates it is impossible to provide any accurate estimates and the best we can do is to illustrate some possible scenarios for one temperature.

The numbers in Table 8 can be compared directly to conventional gas fields and some examples are gien in Fig. 18 above. We have not looked into the monthly variations in Kvitebjørn. The gas treatment plant for Troll gas is located at Kollsnes outside Bergen in Norway. Troll is the largest Norwegian gas field and the guarantist for gas deliveries to Europe according to contracts. Kvitebjørn is one of the smaller fields which is also transported to Kollsnes and treated in a separate gas treatment plant there. Whether the monthly variations are related to balance and agreed delivery rate to Europe or there are other reasons is not known. It is still one of many gas producing fields offshore Norway. Data for all of them in the same format as the data plotted in Fig. 18 is openly available.^67^

**Table 8:** Prod is production rates (billion standard CH_4_ m^−2^ per month) as function of injection gas efficient distribution area (m^2^) and liquid diffusivity coefficients in eqn (21) for a temperature of 274 K

*D*_liq_ area	1 × 10^−8^ m^2^ s^−1^	5 × 10^−8^ m^2^ s^−1^	1 × 10^−7^ m^2^ s^−1^	5 × 10^−7^ m^2^ s^−1^	1 × 10^−6^ m^2^ s^−1^	5 × 10^−6^ m^2^ s^−1^
	Prod	Prod	Prod	Prod	Prod	Prod
1	6.54 × 10^−5^	3.26 × 10^−4^	6.54 × 10^−4^	3.26 × 10^−3^	6.54 × 10^−3^	3.26 × 10^−2^
5	3.38 × 10^−4^	1.63 × 10^−3^	3.38 × 10^−3^	1.63 × 10^−2^	3.38 × 10^−2^	1.63 × 10^−1^
10	6.54 × 10^−4^	3.26 × 10^−3^	6.54 × 10^−3^	3.26 × 10^−2^	6.54 × 10^−2^	3.26 × 10^−1^
50	3.38 × 10^−3^	1.63 × 10^−2^	3.38 × 10^−2^	1.63 × 10^−1^	3.38 × 10^−1^	1.63

Although much of the example conditions are related to a limited region of the Black Sea the total amount of hydrates in the Black Sea between Bulgaria and Romania is significant and can supply with gas for many years the EU (2021 gas consumption of 396.6 bcm). Exact how many years depends on the way the hydrates are produced and efficiency. Assuming the GHD in the REEZ with the same reserves as the GHD in the BEEZ the two largest GHDs in the Danube Fan are with >6000 bcm gas. Therefore 15 years of gas to EU may be as good a guess as any other based on what we know from current seismic information and other data available.

The CO_2_ storage capacity per m^3^ of CH_4_ hydrate filled sediment under the local conditions and typical hydrate saturations is in the order of 110 kg. To put this number into perspective we can compare to Utsira storage of gas separated from the Slepner field in the North Sea which is a million ton CO_2_ per year, or 2740 ton CO_2_ per 24 hours. This is even small compared to CO_2_ from a full scale gas power plant. The trick at Utsira injection is to bring the condition of the CO_2_ injection close to critical point for a mixture of CO_2_ and CH_4_, *i.e.*, small amounts of CH_4_ is added to the CO_2_ in order to create the high permeability of an injection gas close to critical point. CO_2_/N_2_ injection into Black Sea hydrates facilitate from the added supercritical N_2_ and potentially some CH_4_. With the limited hydrate saturation and a typical porosity of 0.45 in the Black Sea it is expected that CO_2_/N_2_ injection into Black Sea hydrates is expected to manage at least same rates as injection into Utsira. These numbers are based on injection well at Utsira. As discussed elsewhere^2^ low cost drilling technology can make it economically feasible to drill several injection wells and distribute the injection to increase injection capacity. It is therefore technically feasible to reach CO_2_/N_2_ injection rate capacities comparable to established aquifer storage projects and higher.

In contrast to for instance pressure reduction the mechanism is on nano scale and formation of new CO_2_ dominated hydrate happens close to *in situ* CH_4_ hydrates. Within the level of information presently available on Black Sea hydrates there is no reason to believe in anything less than the total recovery of the *in situ* CH_4_ gas hydrate. This is particularly true of the relatively high amount of free water in the pores that can create new hydrate and serve as heat sources for CH_4_ hydrate dissociation.

## 9. Discussion

The substantial variation of experimental designs in open literature reports on CO_2_/CH_4_ swapping using either pure CO_2_ or CO_2_/N_2_ mixtures makes it challenging to review and compare experimental results. The reason is the differences in important kinetic parameters in the different experimental setups. Examples are differences in porosity, differences in injection rates, differences in pore flow characteristics, and efficient free pore water/injection gas contact area. These are just a few important differences from a long list. On top of this, there are frequently experimental boundary conditions that interfere with exchange mechanisms. One example of this is experiments conducted at a constant temperature. As discussed in this work, and many other papers referred to in this paper, a dominating mechanism for the CO_2_/CH_4_ swapping in liquid water range of temperatures is that released heat from the formation of hydrate from free pore water and injection gas releases enough heat to dissociate *in situ* CH_4_ hydrates. Exchanging heat between the hydrate-filled sediment and a cooling section then obviously disturbs the natural impact of the heat delivered from the new hydrate formation gas and free water, and the *in situ* CH_4_ hydrates. Conducting the swapping experiments in a container made of heat-insulating material also induces bias as compared to natural surroundings. In a significant number of experimental papers important details are missing, often details of importance for the CO_2_/CH_4_ swapping mechanism. For these reasons, as well as several additional reasons, we excluded reviews of our experiments along with all other published experiments.

There is also the danger of reviews contributing to bringing errors and possible inadequate models further to a wider audience. A few examples of this can be found in the review paper by Koh *et al.*^91^ The pressure-temperature hydrate stability limits for CO_2_ in their Fig. 23 are not following recent experimental data, as illustrated in Fig. 24 below. Another example is the equations of Kim and Bishnoi^65^ listed on the right column of page 117 below the 4th paragraph in ref ^91^. This is an empirical equation based on experiments with controlled stirring without sediments and no coupling between the various kinetic contributions to phase transition kinetics as discussed in models like Classical Nucleation Theory (see Section 4 for a discussion and references) and more advanced models. The list of examples can be very long but limiting ourselves to a final example here then we can consider the equation below the first paragraph on the right-hand side of page 119 (ref. 91) due to Anderson *et al.*^92^ This equation does not apply to realistic pore sizes in natural sediments. Pores sizes beyond nanoscale pore sizes will have a region close to mineral surfaces for which water structures are very extreme. The water density in the first adsorbed layer of water towards a mineral surface can typically be up to 3 times liquid water density (see Kvamme *et al.*^1^ and references in that paper, including references to fundamental experiments) but has a limited range in the order of 2 to 3 nm from the mineral surface before water density is like regular water. These nano-scale effects are critically important in the nucleation of hydrate toward mineral surfaces. And it also prevents hydrate from sticking directly to mineral surfaces. Except for these effects most of the liquid water in pore volume, in realistic natural gas hydrate-filled sediments, is liquid water. These three examples are not meant as any discredit of the review paper by Koh *et al.*^91^ but rather a warning that reviews of experiments and analyses from other research groups always can involve an element of risk of spreading information and data which are not correct and/or at least may need further details on limitations that are not provided in the review.

**Fig. 27:**
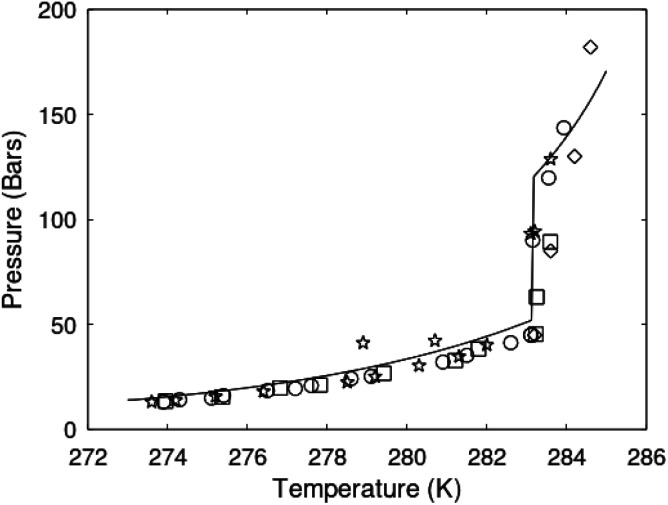
Calculated pressure temperature hydrate stability limits for pure CO_2_ hydrate (solid) as compared to experimental data for CO_2_ hydrate from Herri *et al.*^93^ (circles), Takenouchi and Kennedy^94^ (diamonds), Ohgaki *et al.*^95^ (blue squares) and Fan and Guo^96^ (pentagrams).

Frequently the CO_2_ hydrate stability limits are plotted wrongly in the open literature. There is no reason to speculate on the reasons for that within the focus of this paper but the lack of a steep change in the stability limits practically means that a CO_2_ density change to a higher density is omitted then. Calculated hydrate stability limits (solid curve) are compared to several experimental studies in Fig. 27 below. It is a non-equilibrium system so there is no reason to look for specific points of multiple-phase coexistence as they may not even exist as a stable point in a Gibbs free energy minimum consideration. This is in contrast to an equilibrium situation in which there are of course well defined equilibrium points. The solid line is simply defined and calculated by the conditions of hydrate in equilibrium with liquid water and CO_2_. Higher density for CO_2_ leads to lower fugacity coefficients and then the lower chemical potential for CO_2_ as a separate phase. Higher pressure is therefore needed for the CO_2_ to prefer the hydrate phase in the dense CO_2_ region. This density shift will also of course appear in all mixtures of CO_2_ with other components.

The reason that we have not used a hydrate reservoir simulator to obtain a better perspective of swapping profiles in 2D or 3D modeling is that there are no available hydrate reservoir simulators that have a realistic swapping mechanism incorporated. It is far beyond the scope of this work to give a review of hydrate reservoir simulators. RetrasoCodeBright^34–39^ has a structure that permits the inclusion of kinetic models like the one in eqn (26) but at this stage, there is no budget to pay for the implementation although it will be a similar strategy that was used when implementing a slightly different kinetic model for CH_4_/CO_2_ swapping.^38^

As discussed earlier the technology needed is conventional petroleum technology. The primary focus in future research should therefore be devoted to optimizing the surfactant mixture. To avoid surfactant agglomeration the search is for low MW surfactants, and a mixture with ethanol to optimize the interface effect through the high mobility of ethanol with water.

While N_2_ is used as the primary additive for increasing injection gas permeability air can also be used for simplicity. Thermodynamically the difference between air and N_2_ is not even significant in the thermodynamic analysis conducted in this study.

## 10. Conclusions

In this work, we have mapped the hydrate distributions in the Black Sea between Bulgaria and Romania with the perspective of commercial production of natural gas from these hydrates. Thermal stimulation (steam or other methods) may not be economically feasible and substantial amounts of heat will be listed to other things than hydrate production. Adding chemicals, like for instance thermodynamic hydrate inhibitors, is also expensive and efficiency will be reduced by dilution in pore water for the relatively low hydrate saturations. Two mechanisms can dissociate hydrate during pressure reduction. The establishment of a temperature gradient due to the Joule–Thomson effects generates a low-temperature heat supply and questions are raised on the efficiency of this in breaking hydrogen bonds. Long-term cooling of surrounding sediments is another challenge. Extraction of fluids from hydrate-filled sections will lead to an inflow of fluids from neighboring sediments. These fluids may be under-saturated with natural gas and chemical work-induced hydrate dissociation may occur. These are, however, kinetically slow processes. In summary pressure reduction may be questionable as a feasible production method for Black Sea hydrates.

The hydrate distributions and hydrate saturations make it very feasible for combined storage of CO_2_ in the form of hydrate and the associated release of CH_4_. Injection of CO_2_ and CO_2_/N_2_ mixtures is easy and follows conventional technology. Released CH_4_ will migrate upwards and will be trapped by the sealing structures. Logistics of where to put producing wells is also fairly standard petroleum technology.

The total commercial value of injecting CO_2_/N_2_, CO_2_/air, or flue gas with a CO_2_ content of at least 30 mol% is the combination of storing CO_2_ at a price related to the level of CO_2_ tax, and the value of the produced CH_4_. Cracking the produced gas with steam is an additional option in which the separated CO_2_ from the cracking is returned to the hydrate-filled sediments and Hydrogen is used for local energy production or transport.

There is no reason, at least at this stage, to search for alternatives to N_2_ or air as the permeability-promoting additive to CO_2_. The second additive is added in small portions – typically from ppm and up. The purpose of this additive is to promote fast massive hydrate formation from injection gas (CO_2_/N_2_) while still reducing hydrate blocking of the pores. For this purpose, we seek a mixture of ethanol and a classical low molecular-weight surfactant. In the long run, we seek an environmentally friendly surfactant.

Based on reasonable assumptions and approximations it is possible to reach commercial natural gas for the hydrates in the Black Sea. And given the combined CO_2_ storage value and the value of the produced gas, the total value may even be higher than for conventional natural gas.

An extra value can be added by combining the CH_4_/CO_2_ swap with steam cracking of the produced CH_4_. This leaves H_2_ as the only export product for clean energy use. In a wider context this particular extension can open up for population of remote permafrost areas since it is basically a stand-alone solution in which the steam cracking for the H_2_ production generates the CO_2_ needed for the cycle.

## Conflicts of interest

There are no conflicts of interests.

## Supplementary Material
